# Solvent Polarity as a Selective Modulator of the First Hyperpolarizability in Para-Substituted Azo–Carbazole Dyes

**DOI:** 10.1021/acsomega.5c12352

**Published:** 2026-04-29

**Authors:** Murilo B. M. Ferreira, Herbert C. Georg, Marcos A. Castro, Tertius L. Fonseca

**Affiliations:** Instituto de Física, 67824 Universidade Federal de Goiás, Goiânia, Goiás 74690-900, Brazil

## Abstract

Azo–carbazole monolithic dyes are promising candidates for photonic and optoelectronic applications due to their tunable push–pull character and strong nonlinear optical response. Here, we present a systematic density functional theory (DFT)/time-dependent DFT investigation of seven para-substituted azo–carbazoles (AmACzE, MACzE, HACzE, FACzE, AACzE, CACzE, and NACzE), aimed at elucidating how solvent polarity modulates the first hyperpolarizability (β_HRS_) using long-range corrected hybrid functionals combined with a polarizable continuum model. Our results demonstrate that solvent polarity does not act as a universal enhancer of nonlinear response, but rather as a selective amplifier whose efficiency is governed by the intrinsic charge-transfer capability of the chromophore. Electron-withdrawing substituents (F, COCH_3_, CN, and NO_2_) establish a well-defined push–pull axis, leading to large ground-to-excited-state dipole moment variation (Δμ) and high β_HRS_ values, reaching up to 115 × 10^–30^ esu for NACzE in water. In contrast, donor-substituted derivatives (NH_2_, OCH_3_, and OH) exhibit enhanced local polarization but a limited Δμ, indicating a predominantly localized excited-state character, even in highly polar environments. By correlating β_HRS_ with Δμ within a two-state framework, this work establishes Δμ as a quantitative descriptor controlling both the magnitude and the solvent sensitivity of the nonlinear response. These findings provide clear design guidelines for azo–carbazole-based NLO materials, demonstrating that efficient second-order response arises from the synergistic combination of strong acceptor substitution and polar environments, while also defining the intrinsic limits of solvent-induced enhancement.

## Graphical abstract

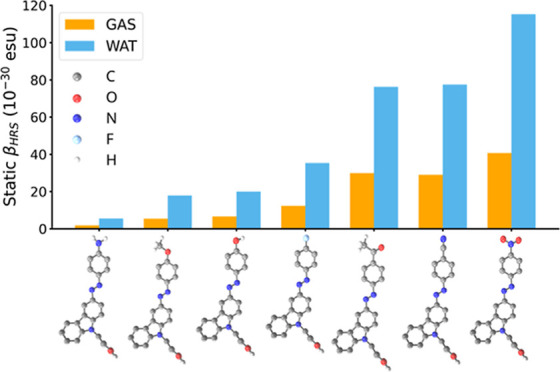

## 1 Introduction

Organic nonlinear optical (NLO) materials play a central role in modern photonic technologies, including frequency conversion, optical modulation, and holographic data storage.^1^ Among them, azo–carbazole-based chromophores have attracted considerable attention due to their structural rigidity, photoresponsiveness, and tunable electronic properties, which make them particularly suitable for rewritable and polarization-multiplexed holographic applications,^2–6^ as well as for NLO applications.^7–9^ Their nonlinear optical response can be rationalized in terms of donor–acceptor substitution and intramolecular charge transfer, strategies that have long guided the molecular design of efficient second-order NLO systems.^10^

Subsequent advances in materials engineering have led to the development of composite films with high optical transparency,^4^ a key attribute to minimize absorption losses and extend the spectral operating window in recording and readout processes. In another approach, the synthesis of specifically designed copolymers enabled improved preservation stability and rewritability of holograms, overcoming limitations related to photochemical fatigue and ensuring consistent performance over multiple cycles.^5^ More recently, the use of azo–carbazole films in polarization-multiplexed holograms has demonstrated remarkable progress in terms of information density and application versatility, enabling everything from high-capacity optical data storage to new display formats, such as dynamic displays and depth-selective three-dimensional projections.^6^

In addition to contributions aimed at holographic applications, fundamental studies with binary carbazole–azo compounds have shown that combining the electron-donor unit of carbazole with a functionalized azo fragment yields highly polarizable molecular architectures with strong coupling between charge-transfer (CT) states and π–π* excitations. This configuration provides significant nonlinear optical responses, including high two-photon absorption and hyperpolarizability values, critical properties for applications in organic photonics.^8^ Complementarily, the analysis of azo–carbazole derivatives designed for diffraction grating recording confirmed, through semiempirical calculations and experimental measurements, a direct correlation between ground-state dipole moments, hyperpolarizabilities, and second-order susceptibilities, reaching χ^(2)^ values as high as 43.2 pm V^–1^.^8^

Taken together, these results show that the advanced photonic performance of azo–carbazole derivatives arises both from molecular design and from the exploitation of their rich CT physics. At the same time, they highlight the need to further investigate long-term stability, synthetic scalability, and integration with hybrid systems; essential factors for establishing these materials as building blocks for emerging photonic technologies.

Beyond the molecular architecture, the surrounding environment is known to modulate the optical response of organic chromophores. Solvent polarity, in particular, is frequently invoked as a key factor enhancing CT stabilization and, consequently, nonlinear optical efficiency. Several studies have demonstrated that solvent polarity can significantly modulate the nonlinear optical responses of organic chromophores.^11–23^ This behavior has been reported for a wide range of π-conjugated systems, including azo-based chromophores with strong acceptor units, push–pull phenylpolyenes,^16^ retinal derivatives,^17^ bifoto cromophores,^18^ and topologically nontrivial systems such as twisted Möbius annulenes.^19^ In these systems, polar environments generally enhance both static and dynamic first hyperpolarizabilities, although the magnitude of the effect strongly depends on the molecular architecture and the nature of the excited state. Notably, studies employing both implicit and explicit solvation models have shown that solvent effects may range from substantial amplification to marginal modulation depending on the balance between charge-transfer character, oscillator strength, and electronic delocalization.

Despite this extensive body of work, solvent polarity is most often treated as a generic amplification factor, while the existence of intrinsic molecular limits to solvent-induced enhancement is rarely addressed in a systematic and quantitative manner. In particular, for azo carbazole-based chromophores, which are widely employed in holographic and photonic applications, the interplay between substituent-controlled CT efficiency and dielectric stabilization remains poorly understood. It is still unclear whether polar environments can compensate for intrinsically inefficient push–pull architectures or whether solvent-induced enhancement is fundamentally constrained by molecular electronic structure.

In this work, we address this gap by performing a systematic density functional theory (DFT)/time-dependent DFT (TDDFT) investigation of seven para-substituted azo–carbazole monolithic dyes, spanning electron-donating to strongly electron-withdrawing substituents and examining their optical and nonlinear optical responses across solvents of increasing polarity [dichloromethane (DCM), acetone (ACE), methanol (MET), dimethyl sulfoxide (DMSO), and water (WAT)]. These monolithic dyes were selected because they are experimentally validated chromophores for rewritable holography, enabling a direct connection between theoretical analysis and device-relevant environments. By correlating absorption spectra, first hyperpolarizabilities, depolarization ratios (DRs), and ground-to-excited-state dipole moment variations, we explicitly demonstrate that solvent polarity acts as a selective amplifier rather than a universal enhancer of nonlinear response. This approach allows us to identify clear physical limits for solvent-assisted NLO enhancement and to establish the ground- to excited-state dipole moment variation as a quantitative descriptor governing both the magnitude and the solvent sensitivity of the first hyperpolarizability. The insights derived from this analysis provide physically grounded design guidelines for the rational development of efficient azo-carbazole-based nonlinear optical materials.

## 2 Computational Methods

To assess solvent effects, geometry optimizations and excited-state properties were computed both in the gas phase (GAS) and in solution using the polarizable continuum model (PCM), in which the solvent is represented as a dielectric continuum characterized by its permittivity.^24^ PCM was employed to isolate dielectric polarization effects and enable a systematic comparison across solvents of increasing polarity, while specific solute–solvent interactions are beyond the scope of the present work. Solvent effects were treated within the linear-response PCM framework, focusing on general dielectric trends rather than state-specific solvation energies.

The ground-state geometries of all azo–carbazole monolithic dyes were optimized within the framework of DFT using three exchange–correlation functionals. Two long-range corrected hybrid generalized gradient approximation (GGA) functionals, ωB97X-D^25^ and CAM-B3LYP,^26^ were employed, in which the exchange interaction is partitioned into short- and long-range components to improve its asymptotic behavior. In addition, the global hybrid *meta*-GGA functional M06–2X^27^ (Minnesota family), which includes an explicit dependence on the local kinetic energy density, was also used. These functionals were selected due to their well-established reliability in describing ground-state molecular structures.^28,29^ Harmonic vibrational frequency analyses confirmed that all optimized structures correspond to genuine minima on the potential energy surface, as no imaginary frequencies were detected. All computations were performed with the 6-311++G­(d,p) basis set using the GAUSSIAN 16 software package.^30^

From an experimental point of view, hyper-Rayleigh scattering (HRS) provides a reliable experimental approach for determining the second-order nonlinear optical response of organic species in solution.^31–33^ The HRS signal is proportional to an orientational average over the square of a combination of hyperpolarizability tensor components. In typical HRS experiments, the vertically polarized scattered light is collected at an angle of 90° with respect to the incident light direction, and the scattered intensity is proportional to β_HRS_^2^(−2ω; ω,ω), which contains the sum of the ⟨β_ZZZ_^2^⟩ and ⟨β_ZXX_^2^⟩ orientational invariants

$$\beta_{HRS}\left(-2\omega ;\omega ,\omega \right)=\sqrt{\left\langle \beta_{ZZZ}^{2}\right\rangle +\left\langle \beta_{ZXX}^{2}\right\rangle }$$

The invariants can be expressed in terms of the molecular components of the β tensor as follows

$$\left\langle \beta_{ZZZ}^{2}\right\rangle =\frac{1}{7}\sum_{\zeta }^{x,y,z}\beta_{\zeta \zeta \zeta }^{2}+\frac{6}{35}\sum_{\zeta \neq \eta }^{x,y,z}\beta_{\zeta \zeta \zeta }\beta_{\zeta \eta \eta }+\frac{9}{35}\sum_{\zeta \neq \eta }^{x,y,z}\beta_{\eta \zeta \zeta }^{2}+\frac{3}{35}\sum_{\zeta \neq \eta \neq \xi }^{x,y,z}\beta_{\eta \zeta \zeta }\beta_{\eta \xi \xi }+\frac{2}{35}\sum_{\zeta \neq \eta \neq \xi }^{x,y,z}\beta_{\zeta \eta \xi }^{2}$$

$$\left\langle \beta_{ZXX}^{2}\right\rangle =\frac{1}{35}\sum_{\zeta }^{x,y,z}\beta_{\zeta \zeta \zeta }^{2}-\frac{2}{105}\sum_{\zeta \neq \eta }^{x,y,z}\beta_{\zeta \zeta \zeta }\beta_{\zeta \eta \eta }+\frac{11}{105}\sum_{\zeta \neq \eta }^{x,y,z}\beta_{\eta \zeta \zeta }^{2}-\frac{1}{105}\sum_{\zeta \neq \eta \neq \xi }^{x,y,z}\beta_{\eta \zeta \zeta }\beta_{\eta \xi \xi }+\frac{4}{105}\sum_{\zeta \neq \eta }^{x,y,z}\beta_{\eta \zeta \zeta }^{2}$$

Following ref 34, the DR is defined as

$$DR=\frac{\left\langle \beta_{ZZZ}^{2}\right\rangle }{\left\langle \beta_{ZXX}^{2}\right\rangle }$$

DR values range from 1.5 to 9, depending on the molecular symmetry. For octupolar molecules, DR is approximately 1.5; for one-dimensional (1D) or linear push–pull molecules, it reaches about 5; and for purely dipolar molecules, it is close to 9.

Excited-state properties, including vertical excitation energies, absorption spectra, first hyperpolarizabilities, DRs, and ground-to-excited-state dipole moment variations, were computed using TDDFT.^35–37^ These property calculations were primarily carried out using the CAM-B3LYP long-range corrected hybrid functional, which offers an improved description of CT excitations.^38^ Additional calculations employing the ωB97X-D, M06-2X, and B3LYP^39^ functionals were performed for selected systems to validate the robustness of the observed trends and are reported in the Supporting Information. The vertical excitation energies and static and dynamic hyperpolarizabilities (evaluated at incident wavelengths of 798, 1064, 1313, and 1550 nm) were obtained with the 6-311++G­(d,p) basis set. All calculations were performed using the GAUSSIAN 16 software package.^30^ The absorption spectra were generated using the Multiwfn program based on the TDDFT excitation energies and oscillator strengths.^40^

## 3 Results and Discussion

### 3.1 Ground State Equilibrium Geometries

CAM-B3LYP/6-311++G­(d,p) calculations of ground-state bond lengths indicate that all azo–carbazole derivatives adopt a globally rigid backbone geometry, with only minor structural variations induced by solvation (Figure 1 and Tables S1–S7, Supporting Information). These variations are small in absolute magnitude, confirming that dielectric effects do not significantly distort the ground-state molecular framework. Instead, the dominant differences in bond-length patterns arise from the electronic nature of the para-substituent, reflecting an intrinsic substituent-dependent electronic asymmetry across the series. Additional ground-state geometry optimizations using the ωB97X-D and M06-2X functionals were performed for all systems to validate these trends and are reported in Tables S8–S21, Supporting Information. Benchmark comparisons show that CAM-B3LYP, ωB97X-D, and M06-2X yield consistent results for geometric properties. On this basis, CAM-B3LYP was selected for the systematic discussion of ground-state structural features presented in the main text.

**Figure 1:**
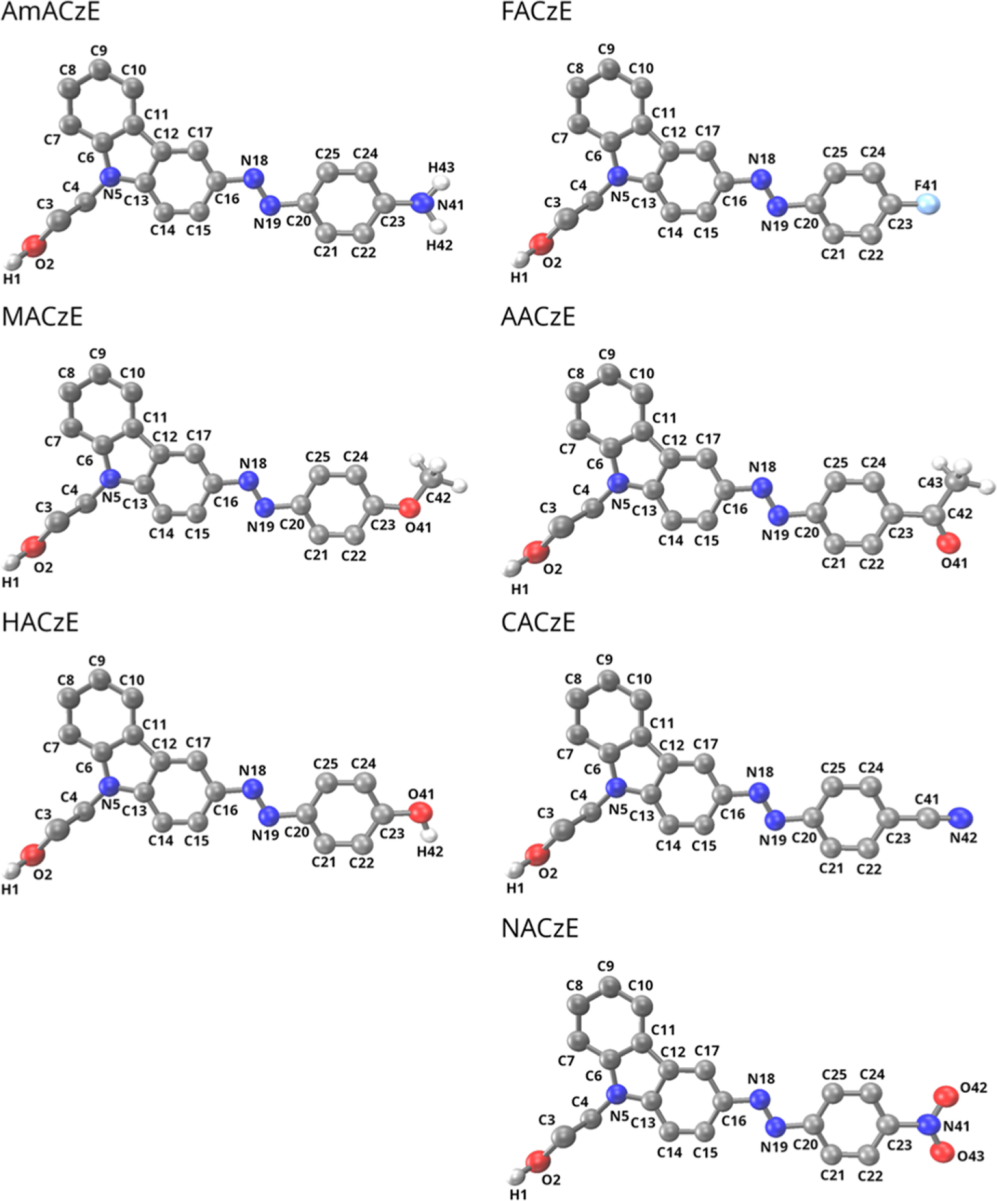
Optimized ground-state geometries and atom numbering of the para-substituted azo–carbazole monolithic dyes investigated in this work. All structures were optimized at the CAM-B3LYP/6-311++G­(d,p) level of theory.

CAM-B3LYP/6-311++G­(d,p) results for the bond-length alternation (BLA) along the phenylazo conjugated pathway are reported in Table 1 for both GAS and the different solvent environments. The BLA was evaluated using the interatomic distances *d*_1_ (C21–C20), *d*_2_ (C22–C21), *d*_3_ (C22–C23), *d*_4_ (C25–C20), *d*_5_ (C25–C24), and *d*_6_ (C24–C23), as defined in Figure 1. It was calculated as the average difference between formally single and double bond lengths along the conjugated pathway according to the following expression

$$BLA=\frac{\left(d_{1}-d_{2}\right)+\left(d_{3}-d_{2}\right)+\left(d_{4}-d_{5}\right)+\left(d_{6}-d_{5}\right)}{4}$$

**Table 1:** Bond-Length Alternation (BLA, in Å) Values for Azo–Carbazole Monolithic Dyes Calculated at the CAM-B3LYP/6-311++G­(d,p) Level in the Gas Phase (GAS) and in Different Solvent Environments: Dichloromethane (DCM), Acetone (ACE), Methanol (MET), Dimethyl Sulfoxide (DMSO), and Water (WAT)

chromophore	GAS	DCM	ACE	MET	DMSO	WAT
AmACzE	0.016	0.018	0.018	0.019	0.019	0.019
MACzE	0.012	0.013	0.013	0.013	0.013	0.013
HACzE	0.012	0.013	0.012	0.013	0.013	0.013
FACzE	0.006	0.007	0.007	0.007	0.007	0.007
AACzE	0.011	0.012	0.012	0.012	0.012	0.012
CACzE	0.013	0.014	0.014	0.014	0.014	0.014
NACzE	0.010	0.012	0.012	0.012	0.012	0.012

In the donor chromophores (AmACzE, MACzE, and HACzE), polar solvation induces subtle but revealing modifications. AmACzE, functionalized with—NH_2_, is the most sensitive: the N–H bonds systematically elongate, while C23–N41 shortens, reinforcing the donor character in solution; in parallel, BLA increases from 0.016 → 0.019 Å, indicating greater electronic localization and loss of effective conjugation. MACzE, with an –OCH_3_ substituent, exhibits shortening of C23–O41 and elongation of C42–O41, evidencing resonant charge transfer from oxygen to the aromatic ring; the increase in BLA (0.012 → 0.013 Å) suggests less homogeneous but more efficient conjugation than in AmACzE. HACzE, bearing –OH, shows shortening of C23–O41 accompanied by elongation of the O–H bond, while maintaining a low BLA (0.012 → 0.013 Å) and preserving global delocalization. In summary, whereas AmACzE undergoes stronger localization and loss of push–pull efficiency, MACzE and HACzE balance resonant donation with structural rigidity.

In the acceptor chromophores (FACzE, AACzE, CACzE, and NACzE), the geometric changes confirm the progressive strengthening of the push–pull character and intramolecular polarization. FACzE, containing fluorine, exhibits minimal variations: the C–F bond slightly elongates (1.348 → 1.353 Å), and BLA increases marginally (0.006 → 0.007 Å), revealing a weak inductive effect and uniform conjugation. AACzE, with –COCH_3_, shows elongation of the carbonyl bond (C=O: 1.211 → 1.217 Å) and an increase in BLA (0.011 → 0.012 Å), indicating enhanced polarization and a tendency toward planarization, favoring electronic delocalization. CACzE, bearing –CN, preserves the rigidity of the nitrile group (C≡N ≈ 1.150 Å) but displays shortening of C13–N5 and C16–N18, along with a slight elongation of N18–N19; the increase in BLA (0.013 → 0.014 Å) reveals density redistribution along the π-chain and reinforcement of the carbazole–azo–phenyl CT axis. NACzE, with –NO_2_, is the most polarized: the shortening of C23–N41 and the concomitant elongation of the N–O bonds intensify the resonant contribution, stabilizing CT states; the rise in BLA confirms its position as the most anisotropic derivative with the highest push–pull efficiency.

The BLA analysis is used here as a qualitative structural descriptor to compare the intrinsic electronic asymmetry across the azo–carbazole series. As shown in Table 1, solvent polarity induces only marginal absolute variations in BLA values, typically on the order of 1 × 10^–4^ Å, indicating that dielectric effects do not significantly alter the ground-state backbone geometry. Instead, the dominant contribution to BLA differences arises from the electronic nature of the para-substituent. Across the series, a clear substituent-dependent trend emerges: donor-substituted systems exhibit slightly larger BLA values, consistent with a more localized ground-state electronic structure, whereas acceptor-substituted derivatives show reduced BLA values, reflecting enhanced π-electron delocalization. These differences reflect intrinsic variations in ground-state electronic distribution rather than solvent-driven structural distortions. Additional BLA parameters calculated with ωB97X-D and M06-2X are reported in Tables S8 and S21 (Supporting Information), confirming the substituent-dependent trends discussed here.

### 3.2 Absorption Spectrum

CAM-B3LYP/6-311++G­(d,p) results for the absorption wavelengths (λ, in nm) and the corresponding oscillator strengths of the azo–carbazole derivatives, both in GAS and in different solvents, are reported in Table 2. Benchmark calculations using B3LYP, M06-2X, ωB97X-D, and CAM-B3LYP for two representative systems (AmACzE and NACzE) are reported in Figure S1 of the Supporting Information. M06-2X, ωB97X-D, and CAM-B3LYP yield consistent qualitative trends for the spectroscopic properties, whereas B3LYP systematically underestimates the excitation energies of the charge-transfer transitions. On this basis, CAM-B3LYP was selected for the systematic discussion of the electronic features presented in the main text.

**Table 2:** Absorption Wavelengths (λ, in nm) and [Corresponding Oscillator Strengths, *f*] Calculated at the CAM-B3LYP/6-311++G­(d,p) Level in the Gas Phase (GAS) and in Different Solvent Environments: Dichloromethane (DCM), Acetone (ACE), Methanol (MET), Dimethyl Sulfoxide (DMSO), and Water (WAT)

chromophore	GAS	DCM	ACE	MET	DMSO	WAT
AmACzE	344.65 [1.1785]	380.28 [1.5008]	384.80 [1.5261]	386.21 [1.5334]	386.96 [1.5371]	387.66 [1.5406]
MACzE	339.13 [1.1200]	368.45 [1.4214]	371.94 [1.4467]	373.00 [1.4540]	373.57 [1.4578]	374.09 [1.4613]
HACzE	335.61 [1.0844]	364.72 [1.4101]	368.20 [1.4373]	369.28 [1.4452]	369.84 [1.4493]	370.35 [1.4529]
FACzE	333.20 [0.9871]	360.48 [1.3122]	363.62 [1.3411]	364.57 [1.3496]	365.08 [1.3540]	365.55 [1.3581]
AACzE	347.59 [1.1779]	379.26 [1.4864]	382.58 [1.5108]	383.57 [1.5177]	384.09 [1.5213]	384.58 [1.5245]
CACzE	349.53 [1.1512]	381.12 [1.4774]	384.44 [1.5048]	385.45 [1.5127]	385.97 [1.5168]	386.44 [1.5205]
NACzE	358.57 [1.1181]	397.92 [1.4365]	401.96 [1.4629]	403.04 [1.4691]	402.63 [1.4640]	403.22 [1.4676]

In GAS, CAM-B3LYP/6-311++G­(d,p) results for azo–carbazole derivatives exhibit absorption between 333 and 359 nm, with oscillator strengths of ∼1.08 and 1.18, except for FACzE, which shows a slightly lower value (0.99). Among the push–pull systems, NACzE (NO_2_) stands out with the most red-shifted transition (358.6 nm), followed by CACzE (349.5 nm) and AACzE (347.6 nm), reflecting the stabilizing effect of electron-withdrawing substituents on the lowest unoccupied molecular orbital (LUMO) orbital. In contrast, chromophores containing donor groups (NH_2_, OCH_3_, and OH) display more blue-shifted absorptions (∼335–345 nm), consistent with the increase in highest occupied molecular orbital (HOMO) energy. These results confirm that even in GAS, the nature of the substituent governs both the spectral position and the intensity of the π–π* electronic transitions.

In the presence of polar solvents, a systematic bathochromic shift is observed in all chromophores (+32 to +45 nm relative to GAS). This effect is most pronounced in the electron-withdrawing derivatives, particularly NACzE, which reaches 403.2 nm in WAT with an oscillator strength of 1.47, standing out as the most environment-sensitive system. Even donor chromophores, such as AmACzE (NH_2_), display significant shifts, although their absorptions remain at shorter wavelengths compared to the push–pull systems. In addition, there is a consistent increase in absorption intensity across all chromophores (Δ*f* increment of ∼29–38%), indicating that solvation differentially stabilizes the ground and excited states, thereby amplifying both the spectral position and the transition intensity. Notably, the results obtained for MET, DMSO, and WAT are very similar, reflecting the sigmoidal dielectric response inherent to the PCM model, which leads to saturation effects at high permittivities.

Figure 2 shows the calculated absorption spectra of AmACzE and NACzE in GAS and polar solvents, displaying a broad absorption band extending from approximately 320 to 550 nm. This band is assigned to π–π transition involving a HOMO → LUMO excitation and reflects the extended conjugation of the carbazole–azo framework, as well as its sensitivity to both solvation and substituent effects. The similar solvent-dependent spectral shifts observed for these two representative donor- and acceptor-substituted chromophores confirm that the trends discussed are general across the series (Figure S2, Supporting Information). The nature of the relevant excited states is further analyzed using natural transition orbitals (NTOs), which provide a more reliable description of CT character than frontier molecular orbitals.^41^ NTOs associated with the lowest-energy optically allowed excitation of AmACzE and NACzE, calculated at the CAM-B3LYP/6-311++G­(d,p) level, are shown in Figure 3. For AmACzE, both the hole and electron NTOs remain largely localized on the carbazole–azo framework, indicating a predominantly localized excited-state character. In contrast, NACzE exhibits a clear spatial separation between the hole, mainly localized on the donor carbazole moiety, and the electron, concentrated on the electron-withdrawing substituent, confirming the pronounced charge-transfer character of the excited state. In addition, to ensure completeness with respect to the frontier-orbital analysis, we have included the corresponding HOMO and LUMO isosurface plots in the Supporting Information for the representative systems AmACzE and NACzE (Figure S3).

**Figure 2:**
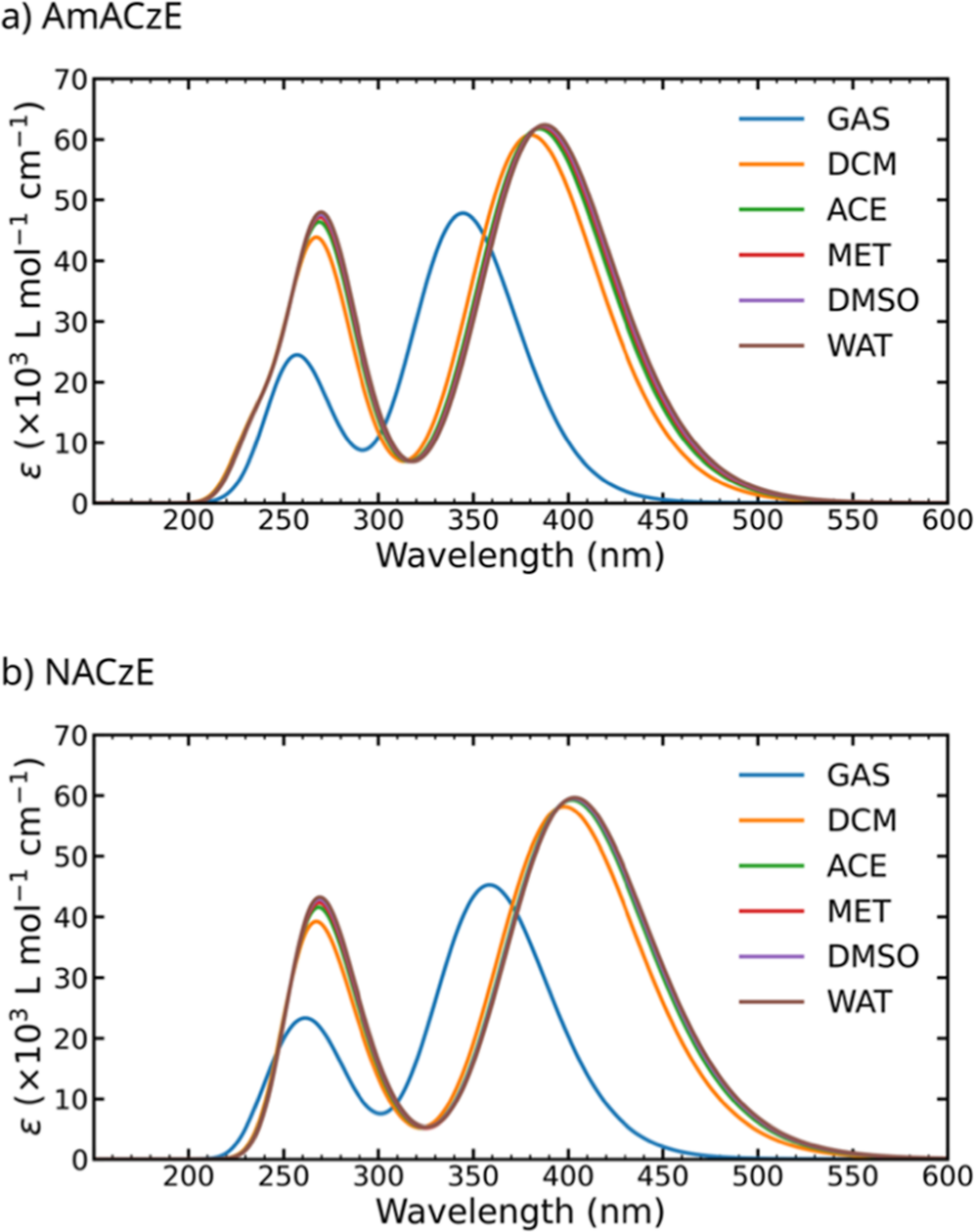
Electronic absorption spectra of AmACzE and NACzE calculated at the CAM-B3LYP/6-311++G­(d,p) level in the gas phase (GAS) and in different solvent environments: dichloromethane (DCM), acetone (ACE), methanol (MET), dimethyl sulfoxide (DMSO), and water (WAT).

**Figure 3:**
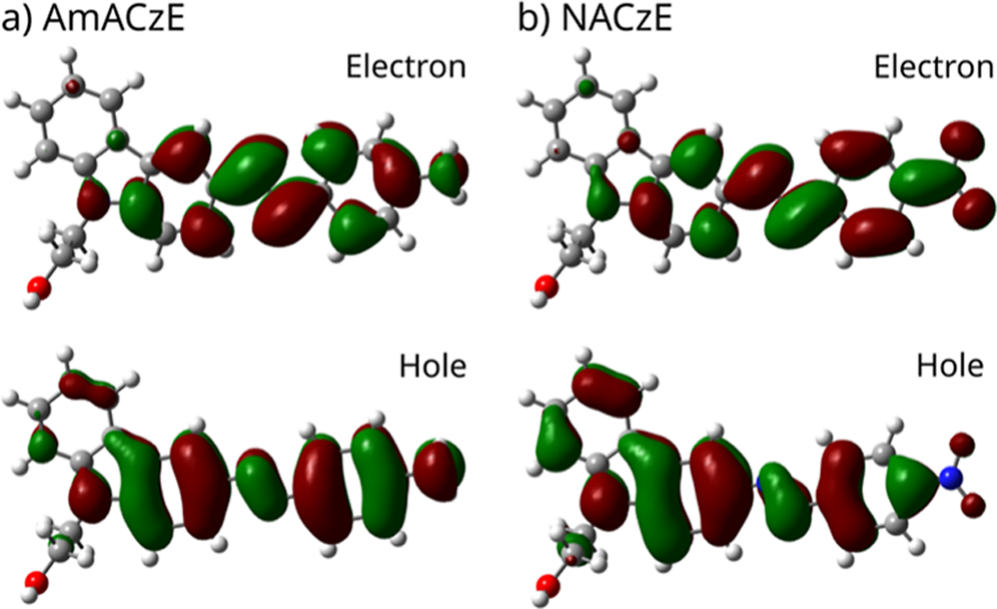
Natural transition orbitals (hole and electron) for the lowest-energy optically allowed excitation of AmACzE and NACzE calculated at the CAM-B3LYP/6-311++G­(d,p) level in water (WAT). AmACzE displays largely localized NTOs, whereas NACzE shows a pronounced spatial separation between hole and electron, characteristic of an excited-state charge-transfer transition.

In PMMA films, the absorption maxima are further red-shifted, ranging from 394 to 440 nm^2^ The acceptor chromophores retain the highest values: NACzE (440 nm), CACzE (426 nm), and AACzE (415 nm). These results are consistent with theoretical predictions, which indicate more pronounced shifts for push–pull systems, confirming their strong intramolecular CT character. In contrast, the donor derivatives (NH_2_, OCH_3_, and OH) absorb between 394 and 410 nm, with AmACzE occupying an intermediate position (410 nm), higher than MACzE and HACzE, but still below the nitro-, cyano-, and aceto-substituted systems. It should be noted that the calculated absorption wavelengths are not expected to quantitatively reproduce experimental values measured in PMMA, due to the different nature of the environment (solid-state polymer matrix versus solution). In this context, the comparison with experiment is intended to be qualitative, focusing on relative trends and chromophore ranking rather than absolute excitation energies.

### 3.3 First Hyperpolarizability

CAM-B3LYP/6-311++G­(d,p) results for the static and dynamic Hyper–Rayleigh Scattering (HRS) first-order hyperpolarizabilities (β_HRS_, in 10^–30^ esu) of the azo–carbazole chromophores in GAS and in different solvent environments are reported in Tables 3 and 4, respectively. Four representative near-infrared (NIR) wavelengths (798, 1064, 1313, and 1550 nm) were considered for the dynamic response. Benchmark β_HRS_ calculations (Table S22, Supporting Information) show that CAM-B3LYP provides a balanced description of both static and dynamic hyperpolarizabilities, avoiding the severe overestimation observed with B3LYP while maintaining consistent charge-transfer trends comparable to those obtained with M06-2X and ωB97X-D.

**Table 3:** Static HRS First Hyperpolarizability (β_HRS_/10^–30^ esu) Results for Azo–Carbazole Monolithic Dyes Calculated at the CAM-B3LYP/6-311++G­(d,p) Level in the Gas Phase (GAS) and in Different Solvent Environments: Dichloromethane (DCM), Acetone (ACE), Methanol (MET), Dimethyl Sulfoxide (DMSO), and Water (WAT)

chromophore	GAS	DCM	ACE	MET	DMSO	WAT
AmACzE	1.75	4.60	5.15	5.33	5.43	5.53
MACzE	5.41	15.18	16.83	17.37	17.65	17.92
HACzE	6.60	17.24	18.94	19.48	19.77	20.04
FACzE	12.30	30.73	33.50	34.38	34.85	35.29
AACzE	29.92	67.71	72.98	74.62	75.48	76.30
CACzE	29.01	68.72	74.14	75.83	76.71	77.54
NACzE	40.70	102.01	110.72	113.34	113.81	115.16

**Table 4:** Dynamic HRS First Hyperpolarizability (β_HRS_/10^–30^ esu) for Azo–Carbazole Monolithic Dyes Calculated at the CAM-B3LYP/6-311++G­(d,p) Level in the Gas Phase (GAS) and in Different Solvent Environments: Dichloromethane (DCM), Acetone (ACE), Methanol (MET), Dimethyl Sulfoxide (DMSO), and Water (WAT)

chromophore	GAS	DCM	ACE	MET	DMSO	WAT
λ = 798 nm						
AmACzE	1.97	3.53	3.47	3.42	3.67	3.46
MACzE	6.79	13.28	13.06	12.87	13.76	13.03
HACzE	8.28	15.25	14.90	14.67	15.64	14.81
FACzE	15.17	27.21	26.56	26.15	27.79	26.37
AACzE	36.89	63.45	62.18	61.33	64.71	61.79
CACzE	35.88	63.48	62.00	61.07	64.59	61.49
NACzE	51.13	97.19	95.67	94.37	99.07	94.48
λ = 1064 nm						
AmACzE	2.12	3.86	3.78	3.73	4.02	3.78
MACzE	7.71	15.29	15.03	14.82	15.87	15.00
HACzE	9.39	17.52	17.12	16.85	18.00	17.02
FACzE	17.05	30.95	30.20	29.72	31.64	29.98
AACzE	41.50	72.63	71.14	70.14	74.14	70.69
CACzE	40.46	72.75	70.99	69.89	74.06	70.39
NACzE	58.17	113.25	111.47	109.90	115.56	110.02
λ = 1313 nm						
AmACzE	2.34	4.33	4.24	4.18	4.52	4.24
MACzE	9.02	18.24	17.93	17.67	18.96	17.89
HACzE	10.98	20.84	20.37	20.04	21.45	20.24
FACzE	19.70	36.34	35.45	34.87	37.19	35.19
AACzE	48.10	86.18	84.36	83.12	88.08	83.81
CACzE	47.04	86.46	84.29	82.93	88.09	83.55
NACzE	68.40	137.69	135.51	133.53	140.71	133.67
λ = 1550 nm						
AmACzE	2.95	5.65	5.53	5.43	5.93	5.52
MACzE	12.52	26.51	26.06	25.67	27.67	26.02
HACzE	15.17	30.07	29.37	28.88	31.05	29.21
FACzE	26.55	51.00	49.69	48.83	52.32	49.32
AACzE	65.74	124.91	122.09	120.15	128.10	121.27
CACzE	64.79	125.89	122.43	120.27	128.59	121.28
NACzE	96.72	212.54	209.15	205.83	218.29	206.09

A clear substituent-dependent hierarchy emerges across the series. Chromophores bearing electron-withdrawing groups (FACzE, AACzE, CACzE, and NACzE) exhibit the largest β_HRS_ values in all environments, consistent with the stabilization of CT excited states promoted by acceptor substituents. Within this group, NACzE represents the upper bound of the series, displaying the strongest nonlinear response and the highest sensitivity to solvent polarity. Its static β_HRS_ value increases from 40.70 × 10^–30^ esu in GAS to 115.16 × 10^–30^ esu in WAT, corresponding to nearly a 3-fold enhancement. AACzE and CACzE show nearly identical behavior, both in absolute magnitude and in solvent dependence, confirming that carbonyl and cyano substituents induce comparable CT efficiencies. FACzE follows the same qualitative trend, albeit with reduced amplitude, reflecting the weaker electron-withdrawing character of fluorine.

In contrast, donor-substituted chromophores (AmACzE, MACzE, and HACzE) display much smaller nonlinear responses, with static β_HRS_ values ranging from 1 to 7 × 10^–30^ esu in GAS and reaching at most 20 × 10^–30^ esu in polar solvents. Although these systems exhibit large relative increases upon solvation, often exceeding 200%, their absolute β_HRS_ values remain approximately 1 order of magnitude lower than those of the acceptor derivatives. This behavior indicates that electron-donating substituents enhance local polarization but intrinsically limit directional charge transfer, constraining the nonlinear response even in highly polar environments.

Across the entire series, solvation systematically enhances β_HRS_ when moving from GAS to polar solvents, reflecting the preferential stabilization of CT excited states by dielectric polarization. For strongly accepting systems, solvent effects reinforce an already efficient push–pull architecture, nearly doubling the response, whereas in donor chromophores, solvation primarily amplifies modest intrinsic responses. Importantly, the enhancement saturates at high dielectric constants, as evidenced by the very similar β_HRS_ values obtained in MET, DMSO, and WAT, indicating the existence of an upper bound for dielectric-driven amplification within the PCM framework.

It is important to emphasize that in the context of the present study, the solvent does not play a direct functional role in holographic devices. Instead, it is employed as a controlled dielectric model to probe how environmental polarization modulates the electronic structure and nonlinear optical response of azo–carbazole dyes. By systematically varying solvent polarity, we selectively probe the stabilization of CT states and the associated modulation of the first hyperpolarizability. The trends observed in solution are therefore not solvent-specific but rather reflect general dielectric effects that are directly transferable to condensed-phase environments, such as polymer matrices and solid films commonly used in photonic and holographic applications.

The dynamic hyperpolarizabilities show a monotonic increase with wavelength for all chromophores and environments, consistent with electronic dispersion effects as the excitation energy approaches the resonance level (Table 4). This effect is most pronounced in acceptor-substituted systems. For example, NACzE exhibits an increase from 51.13 × 10^–30^ esu at 798 nm to 96.72 × 10^–30^ esu at 1550 nm in GAS and reaches values above 218 × 10^–30^ esu in polar solvents at 1550 nm. AACzE and CACzE show nearly identical dispersion behavior, while FACzE displays intermediate growth. These results indicate that in the near-infrared regime, push–pull chromophores with strong acceptor substituents are more sensitive to frequency variation, reinforcing their suitability for wavelength-tunable nonlinear photonic applications such as frequency conversion, optical modulation, and infrared second-harmonic generation. Donor-substituted chromophores also exhibit wavelength-dependent enhancement but with smaller growth factors, reflecting the weaker contribution of CT states.

Overall, these results demonstrate that molecular design and environmental polarization act synergistically in determining the magnitude of the nonlinear optical response but within well-defined physical limits. Solvent polarity acts as a dielectric amplifier rather than a universal enhancer, selectively reinforcing the response of chromophores with intrinsically efficient charge-transfer architectures.

### 3.4 Depolarization Ratios

Table 5 reports the static DRs for the azo–carbazole chromophores in GAS and in different solvent environments, providing insight into the anisotropy of their nonlinear optical response. A clear substituent-dependent pattern is observed. Chromophores containing strong electron-withdrawing groups (NACzE, CACzE, AACzE, and FACzE) exhibit consistently high DR values, typically in the range 4.2–4.7, indicative of a strongly unidirectional polarization axis associated with efficient charge transfer. NACzE represents the extreme case, reaching DR values close to 4.7 in polar solvents, while AACzE and CACzE display nearly identical anisotropies, consistent with their similar electronic structures.

**Table 5:** Static Depolarization Ratios for Azo–Carbazole Monolithic Dyes Calculated at the CAM-B3LYP/6-311++G­(d,p) Level in the Gas Phase (GAS) and in Different Solvent Environments: Dichloromethane (DCM), Acetone (ACE), Methanol (MET), Dimethyl Sulfoxide (DMSO), and Water (WAT)

chromophore	GAS	DCM	ACE	MET	DMSO	WAT
AmACzE	2.00	2.19	2.23	2.25	2.26	2.26
MACzE	3.24	3.61	3.64	3.65	3.66	3.67
HACzE	3.57	3.88	3.90	3.91	3.91	3.92
FACzE	4.16	4.32	4.33	4.33	4.34	4.34
AACzE	4.53	4.56	4.56	4.56	4.56	4.56
CACzE	4.53	4.59	4.58	4.58	4.58	4.58
NACzE	4.60	4.67	4.67	4.67	4.67	4.67

In contrast, donor-substituted chromophores show significantly lower and more variable DR values. AmACzE exhibits the most isotropic response, with DR values close to 2.0, indicating an inefficient and multidirectional charge redistribution. MACzE and HACzE occupy intermediate positions, with DR values ranging from approximately 3.2 to 3.9, reflecting a mixed polarization behavior with partial directionality. This hierarchy confirms that electron-withdrawing substituents promote electronic asymmetry and a well-defined push–pull axis, whereas donor substituents reduce anisotropy by favoring localized excited states.

Solvent effects on the DR are comparatively modest. Polar environments induce small but systematic increases, particularly in donor-substituted systems, where DR values rise by approximately 0.3–0.4 units relative to GAS. In acceptor chromophores, the effect of solvation on DR is negligible, as these systems already possess a highly anisotropic electronic structure. This behavior further supports the view that solvent polarity amplifies intrinsic electronic features rather than alters the fundamental nature of the nonlinear response.

The frequency dependence of DR, summarized in Table 6, reveals that anisotropy generally increases with wavelength for acceptor-substituted chromophores, consistent with the growing dominance of CT contributions under near-resonant conditions. NACzE, AACzE, and CACzE exhibit gradual increases in DR as the wavelength extends from 798 to 1550 nm, reinforcing their unidirectional polarization character. FACzE shows a similar but slightly more pronounced increase. In contrast, AmACzE becomes progressively more isotropic at longer wavelengths, while MACzE and HACzE display only moderate growth, reflecting their limited charge-transfer efficiency.

**Table 6:** Dynamic Depolarization Ratios for Azo–Carbazole Monolithic Dyes Calculated at the CAM-B3LYP/6-311++G­(d,p) Level in the Gas Phase (GAS) and in Different Solvent Environments: Dichloromethane (DCM), Acetone (ACE), Methanol (MET), Dimethyl Sulfoxide (DMSO), and Water (WAT)

chromophore	gas	DCM	ACE	MET	DMSO	WAT
λ = 798 nm						
AmACzE	1.78	2.10	2.15	2.17	2.18	2.19
MACzE	3.42	3.77	3.79	3.80	3.81	3.81
HACzE	3.75	4.04	4.06	4.06	4.07	4.07
FACzE	4.27	4.44	4.45	4.45	4.45	4.45
AACzE	4.60	4.67	4.68	4.68	4.68	4.68
CACzE	4.60	4.68	4.69	4.69	4.69	4.69
NACzE	4.68	4.76	4.77	4.77	4.77	4.77
λ = 1064 nm						
AmACzE	1.68	2.02	2.07	2.09	2.10	2.10
MACzE	3.52	3.87	3.89	3.90	3.91	3.91
HACzE	3.84	4.13	4.14	4.15	4.16	4.16
FACzE	4.33	4.49	4.50	4.50	4.51	4.51
AACzE	4.64	4.71	4.71	4.72	4.72	4.72
CACzE	4.64	4.72	4.73	4.73	4.73	4.73
NACzE	4.71	4.80	4.80	4.80	4.80	4.80
λ = 1313 nm						
AmACzE	1.59	1.93	1.98	2.00	2.02	2.02
MACzE	3.64	3.99	4.01	4.02	4.03	4.03
HACzE	3.95	4.23	4.25	4.25	4.26	4.26
FACzE	4.40	4.56	4.57	4.57	4.57	4.57
AACzE	4.68	4.75	4.76	4.76	4.76	4.76
CACzE	4.69	4.77	4.77	4.77	4.77	4.77
NACzE	4.76	4.84	4.84	4.84	4.84	4.84
λ = 1550 nm						
AmACzE	1.52	1.82	1.87	1.88	1.91	1.90
MACzE	3.88	4.22	4.24	4.25	4.26	4.26
HACzE	4.17	4.43	4.44	4.45	4.46	4.45
FACzE	4.53	4.68	4.69	4.69	4.69	4.69
AACzE	4.76	4.84	4.84	4.84	4.85	4.85
CACzE	4.78	4.85	4.86	4.86	4.86	4.86
NACzE	4.84	4.91	4.92	4.92	4.92	4.92

Taken together, the DR analysis complements the hyperpolarizability results by directly linking the nonlinear response anisotropy to substituent-controlled charge transfer. High DR values correlate with large β_HRS_ responses and strong excited-state polarization, whereas low DR values signal intrinsic limitations in directional charge redistribution. These trends confirm that both the magnitude and the anisotropy of the nonlinear optical response are governed primarily by the molecular electronic structure, with solvent polarity acting as a secondary, amplifying factor.

### 3.5 Relation between Δμ and β_HRS_

Complementarily, the solvent effects on dipole moment values reveal a clear pattern of the selective amplification of electronic polarization. For all chromophores, the excited-state dipole moment systematically increases when going from the GAS to polar solvents, while the ground state remains practically unchanged (Table 7). This behavior indicates that solvation differentially stabilizes excited states with CT character, enhancing the ground-state-to-excited-state dipole moment variation (Δμ). The effect is particularly pronounced in strong acceptor chromophores: NACzE evolves from ∼7.4 au in GAS to ∼10.1 au in WAT, while CACzE and AACzE increase from ∼6.3/5.2 to ∼8.3/7.2 au, respectively. FACzE, with its weaker inductive effect, shows a more moderate growth (3.7 → 5.0 au). In contrast, donor systems display more discrete changes: AmACzE maintains very low values (≈1.0 → 1.35 au), reflecting weak charge transfer, while MACzE and HACzE, although somewhat more sensitive, reach only ∼2.1 and ∼2.6 au in WAT. Taken together, the data confirm that polar solvents act as stabilizing fields that intensify the intrinsic polarization of acceptor derivatives but have a limited impact on donor systems, reinforcing the push–pull hierarchy that governs the nonlinear optical response of the series.

**Table 7:** Results for the Ground-Sate Dipole Moment (μ_es_, in a.u.) and the [Ground-to-Excited-State Dipole Moment Variation (Δμ = μ_es_ – μ_gs_, in a.u.)] Calculated at the CAM-B3LYP/6-311++G­(d,p) Level in the Gas Phase (GAS) and in Different Solvent Environments: Dichloromethane (DCM), Acetone (ACE), Methanol (MET), Dimethyl Sulfoxide (DMSO), and Water (WAT)

chromophore	GAS	DCM	ACE	MET	DMSO	WAT
AmACzE	1.051 [0.271]	1.268 [0.149]	1.301 [0.174]	1.311 [0.184]	1.317 [0.190]	1.322 [0.196]
MACzE	0.732 [0.999]	0.917 [1.389]	0.946 [1.440]	0.955 [1.456]	0.960 [1.464]	0.965 [1.472]
HACzE	1.080 [1.338]	1.353 [1.723]	1.389 [1.768]	1.401 [1.782]	1.407 [1.789]	1.413 [1.796]
FACzE	1.575 [2.207]	1.924 [3.135]	1.963 [3.037]	1.975 [3.165]	1.981 [3.181]	1.987 [3.165]
AACzE	2.131 [3.149]	2.674 [4.288]	2.731 [4.415]	2.748 [4.454]	2.757 [4.474]	2.766 [4.493]
CACzE	3.158 [3.208]	3.771 [4.388]	3.829 [4.516]	3.846 [4.554]	3.855 [4.574]	3.864 [4.593]
NACzE	3.352 [4.150]	4.016 [5.814]	4.080 [5.991]	4.099 [6.043]	4.110 [6.062]	4.120 [6.087]

In acceptor derivatives (FACzE, AACzE, CACzE, NACzE), Δμ is amplified by solvation: it increases from 2.21 au in FACzE (GAS) to 3.16 au in WAT, from 3.15 → 4.49 au in AACzE, from 3.21 → 4.59 au in CACzE, and from 4.15 → 6.09 au in NACzE, as illustrated in Figure 4. The β_HRS_ values follow the same trend: NACzE reaches 115 × 10^–30^ esu in WAT, CACzE and AACzE reach ∼75 × 10^–30^ esu, and FACzE ∼35 × 10^–30^ esu. Here, the Δμ ↔ β_HRS_ correlation is evident: the greater the charge separation in the excited state, the stronger the nonlinear response. NACzE represents the extreme case, combining a high Δμ value with maximum β_HRS_, reflecting its strong electron-accepting character (–NO_2_). This behavior shows that electronic excitation enhances the CT character of the excited state, reinforcing the push–pull character and structural anisotropy already suggested by the geometric parameters and DR.

**Figure 4:**
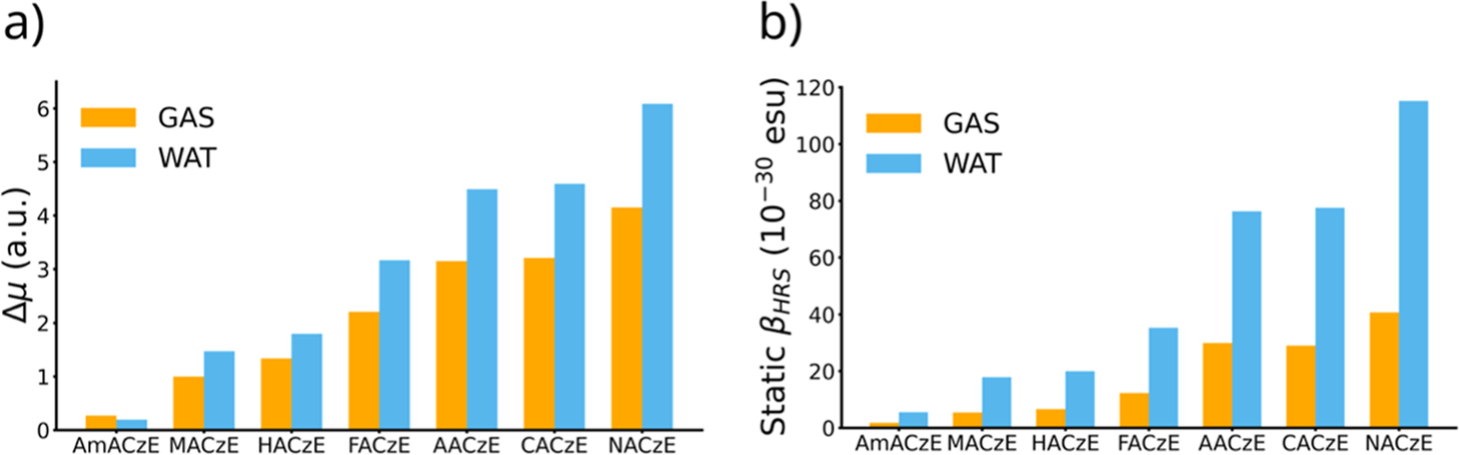
(a) Bar chart comparing the ground-to-excited-state dipole moment variation (Δμ = μ_es_ – μ_gs_) and (b) static β_HRS_ values in the gas phase (GAS) and in water (WAT) for the seven chromophores.

In contrast, the donor derivatives present smaller variations and, in the extreme case of AmACzE, a reduction in Δμ values in polar solvents (Δμ = 0.27 au in GAS, decreasing to 0.20 au in WAT), indicating that electronic excitation redistributes the density in a nearly isotropic manner. MACzE and HACzE, in turn, show moderate increases, with Δμ growing from 0.99 → 1.47 and 1.34 → 1.80 au, respectively, which indicates effective resonance donation, though less directional than in strong acceptors. Consequently, the β_HRS_ values remain low, reaching at most 5–20 × 10^–30^ esu in WAT—1 order of magnitude below the acceptor systems. The correlation is clear: the small dipole variation limits push–pull efficiency, and even when Δμ increases in solvents, β_HRS_ responds only modestly, reflecting the intrinsic restriction in electronic charge transfer.

These results confirm that polar solvation not only amplifies the character of the substituent but also governs the efficiency of charge transfer in the excited state, a critical aspect for controlling dynamic hyperpolarizability and the nonlinear optical response under excitation conditions. From a physicochemical standpoint, the trends observed in Figure 4 are consistent with the two-state model, in which the first-order hyperpolarizability follows the relationship^XX^

$$\beta^{TL}\propto \Delta \mu f_{0}/\Delta E^{3}$$

where Δ*E* and *f*_0_ correspond to the transition energy and the oscillator strength, respectively. The dipole moment variation between ground and excited states thus emerges as the dominant parameter controlling the magnitude of the nonlinear response in the azo–carbazole derivatives, particularly under solvent-induced polarization conditions.

## 4 Conclusions

In this work, we performed a comprehensive DFT/TDDFT analysis of solvent effects on the linear and nonlinear optical properties of a series of para-substituted azo–carbazole monolithic dyes. Although the molecular backbone remains globally rigid upon solvation, subtle substituent-dependent structural and electronic modulations govern the efficiency of charge transfer and, consequently, the nonlinear optical response.

A central result of this study is that solvent polarity does not uniformly enhance the first hyperpolarizability across the series. Instead, solvation acts as a selective amplifier, whose impact is dictated by the intrinsic push–pull character of the chromophore. Electron-donating substituents increase local polarization but promote electronic localization, leading to small Δμ and low β_HRS_ values (≤20 × 10^–30^ esu in WAT), even in highly polar solvents. In contrast, electron-withdrawing substituents progressively reinforce the CT character of the excited state, resulting in large Δμ values and substantially enhanced β_HRS_ responses. Among the systems investigated, NACzE emerges as the most efficient chromophore, combining strong acceptor substitution with polar solvation to reach β_HRS_ values exceeding 115 × 10^–30^ esu.

Importantly, the solvent-induced enhancement was found to saturate at high dielectric constants, indicating the existence of an upper bound for dielectric-driven amplification within the PCM framework. This behavior highlights that solvent polarity alone cannot compensate for intrinsically inefficient CT architectures. The strong correlation observed between Δμ and β_HRS_ across all environments confirms the validity of a two-state model description and establishes Δμ as a key quantitative descriptor for predicting both the magnitude and solvent sensitivity of second-order NLO responses.

Overall, this study provides physically grounded design principles for azo carbazole-based NLO materials: efficient nonlinear performance requires the synergistic combination of strong electron-accepting substituents and polar environments, while donor-substituted systems remain fundamentally limited. By explicitly identifying both the amplification regime and its intrinsic limits, this work offers guidance for rational molecular engineering of solution- and solid-state photonic materials.

## Supplementary Material



## Notes

DFT results for bond lengths and bond-length alternation (BLA) coordinates (in Å) of ACzE molecules in the gas phase and in solution for the ground state, TDDFT-calculated absorption spectra of AmACzE and NACzE in WAT obtained using the B3LYP, M06-2X, ωB97X-D, and CAM-B3LYP functionals, CAM-B3LYP absorption spectra of MACzE, HACzE, FACzE, AACzE, and CACzE calculated in the gas phase and in different solvent environments, frontier molecular orbitals (HOMO and LUMO) of AmACzE and NACzE computed at the CAM-B3LYP/6-311++G­(d,p) level in WAT, and DFT results for the static and dynamic hyper-Rayleigh scattering (HRS) first hyperpolarizabilities of AmACzE and NACzE in water (PDF)

## References

[1] Delaire J. A., Nakatani K. (2000). Linear and Nonlinear Optical Properties of Photochromic
Molecules and Materials. Chem. Rev..

[2] Kinashi K., Fukami T., Yabuhara Y., Motoishi S., Sakai W., Kawamoto M., Sassa T., Tsutsumi N. (2016). Molecular Design of
Azo-Carbazole Monolithic Dyes for Updatable Full-Color Holograms. NPG Asia Mater..

[3] Tsutsumi N., Kinashi K., Ogo K., Fukami T., Yabuhara Y., Kawabe Y., Tada K., Fukuzawa K., Kawamoto M., Sassa T., Fujihara T., Sasaki T., Naka Y. (2015). Updatable
Holographic Diffraction of Monolithic Carbazole-Azobenzene Compound
in Poly­(methyl methacrylate) Matrix. J. Phys.
Chem. C.

[4] Kinashi K., Nakanishi I., Sakai W., Tsutsumi N., Jackin B. J. (2023). Azo-Carbazole
Copolymer-Based Composite Films with High Optical Transparency for
Updatable Holograms. New J. Chem..

[5] Kinashi K., Nakanishi I., Sakai W., Tsutsumi N. (2019). Material Design of
Azo-Carbazole Copolymers for Preservation Stability with Rewritable
Holographic Stereograms. Macromol. Chem. Phys..

[6] Singh S. K., Kinashi K., Tsutsumi N., Sakai W., Jackin B. J. (2024). High-Density
Polarization Multiplexed Holograms Using Azo-Carbazole Films for Diverse
Applications. Opt. Express.

[7] Huang X., Zhong S., Yan X., Ke X., Srisanit N., Wang M. R. (2004). The Synthesis and Nonlinear Optical Property of Carbazole-Azo
Binary Compounds. Synth. Met..

[8] Czaplicki, R. ; Seignon, S. D. ; Kajzar, F. ; Bakasse, M. ; Nizioł, J. ; Bednarz, M. ; Sahraoui, B. Functionalized Azo-Carbazole Compounds for Nonlinear Optical Application. International Conference on Transparent Optical Networks; Institute of Electrical and Electronics Engineers, 2006, pp 283–286..

[9] Nizioł J., Gondek E., Pluciński K. J. (2010). Azo-Carbazole
Dye Chromophore as
Promising Materials for Diffraction Grating Recording. J. Mater. Sci. Mater. Electron..

[10] Oudar J. L., Chemla D. S. (1977). Hyperpolarizabilities
of the Nitroanilines and Their
Relations to the Excited State Dipole Moment. J. Chem. Phys..

[11] Clays K., Persoons A. (1991). Hyper-Rayleigh Scattering
in Solution. Phys. Rev. Lett..

[12] Dehu C., Meyers F., Hendrickx E., Clays K., Persoons A., Marder S. R., Brédas J. L. (1995). Solvent
Effects on the Second-Order
Nonlinear Optical Response of π-Conjugated Molecules: A Combined
Evaluation through Self-Consistent Reaction Field Calculations and
Hyper-Rayleigh Scattering Measurements. J. Am.
Chem. Soc..

[13] Kaatz P., Shelton D. P. (1996). Polarized Hyper-Rayleigh
Light Scattering Measurements
of Nonlinear Optical Chromophores. J. Chem.
Phys..

[14] Huyskens F. L., Huyskens P. L., Persoons A. P. (1998). Solvent
Dependence of the First Hyperpolarizability
of *p*-Nitroanilines: Differences between Nonspecific
Dipole–Dipole Interactions and Solute–Solvent H-Bonds. J. Chem. Phys..

[15] Shalin N.
I., Phrolycheva Y. A., Fominykh O. D., Balakina M. Y. (2021). Solvent Effect on
Static and Dynamic First Hyperpolarizability of Azochromophore with
Tricyanopyrrole Acceptor Moiety. J. Mol. Liq..

[16] Ferrighi L., Frediani L., Cappelli C., Sałek P., Ågren H., Helgaker T., Ruud K. (2006). Density-Functional-Theory
Study of the Electric-Field-Induced Second Harmonic Generation (EFISHG)
of Push–Pull Phenylpolyenes in Solution. Chem. Phys. Lett..

[17] Junior L. A., Colherinhas G., Fonseca T. L., Castro M. A. (2014). Solvent Effects
on the First Hyperpolarizability of Retinal Derivatives. Chem. Phys. Lett..

[18] Quertinmont J., Champagne B., Castet F., Hidalgo Cardenuto M. (2015). Explicit versus
Implicit Solvation Effects on the First Hyperpolarizability of an
Organic Biphotochrome. J. Phys. Chem. A.

[19] Alam M. M., Kundi V., Thankachan P. P. (2016). Solvent
Effects on Static Polarizability,
Static First Hyperpolarizability, and One- and Two-Photon Absorption
Properties of Functionalized Triply Twisted Möbius Annulenes:
A DFT Study. Phys. Chem. Chem. Phys..

[20] Marrazzini G., Giovannini T., Egidi F., Cappelli C. (2020). Calculation of Linear
and Nonlinear Electric Response Properties of Systems in Aqueous Solution:
A Polarizable Quantum/Classical Approach with Quantum Repulsion Effects. J. Chem. Theory Comput..

[21] Fonseca S., dos Santos N. S. S., Georg H. C., Fonseca T. L., Provasi P. F., Coutinho K., Canuto S., da Cunha A. R., Gester R. (2024). Elucidating
the Photophysics and Nonlinear Optical Properties of a Novel Azo Prototype
for Possible Photonic Applications: A Quantum Chemical Analysis. ACS Omega.

[22] Brandão I., Georg H. C., Castro M. A., Fonseca T. L. (2024). Calculation of the
Geometry, Absorption Spectrum, and First Hyperpolarizability of 4,5-Dicyanoimidazole
Derivatives in Solution: A Multiscale ASEC–FEG Study. J. Chem. Phys..

[23] Brandão I., Franco L. R., Fonseca T. L., Castro M. A., Georg H. C. (2017). Confirming
the Relationship between First Hyperpolarizability and the Bond Length
Alternation Coordinate for Merocyanine Dyes. J. Chem. Phys..

[24] Tomasi J., Mennucci B., Cammi R. (2005). Quantum Mechanical
Continuum Solvation
Models. Chem. Rev..

[25] Chai J.-D., Head-Gordon M. (2008). Long-Range Corrected Hybrid Density Functionals with
Damped Atom–Atom Dispersion Corrections. Phys. Chem. Chem. Phys..

[26] Yanai T., Tew D. P., Handy N. C. (2004). A New Hybrid
Exchange–Correlation
Functional Using the Coulomb-Attenuating Method (CAM-B3LYP). Chem. Phys. Lett..

[27] Zhao Y., Truhlar D. G. (2008). The M06 Suite of
Density Functionals for Main Group
Thermochemistry, Thermochemical Kinetics, Nonncovalent Interactions,
Excited States, and Transition Elements: Two New Functionals and Systematic
Testing of Four M06-Class Functionals and 12 Other Functionals. Theor. Chem. Acc..

[28] Rtibi E., Abderrabba M., Ayadi S., Champagne B. (2019). Theoretical
Assessment of the Second-Order Nonlinear Optical Responses of Lindqvist-Type
Organoimido Polyoxometalates. Inorg. Chem..

[29] Al-Yasari A., Van Steerteghem N., El Moll H., Clays K., Fielden J. (2016). Donor–Acceptor
Organo-Imido Polyoxometalates: High Transparency, High Activity Redox-Active
NLO Chromophores. Dalton Trans..

[30] Frisch, M. J. ; Trucks, G. W. ; Schlegel, H. B. ; Scuseria, G. E. ; Robb, M. A. ; Cheeseman, J. R. ; Scalmani, G. ; Barone, V. ; Petersson, G. A. ; Nakatsuji, H. ; Gaussian 16, Revision C.01; Gaussian Inc.: Wallingford, CT, 2016..

[31] Hendrickx E., Clays K., Persoons A. (1998). Hyper-Rayleigh
Scattering in Isotropic
Solution. Acc. Chem. Res..

[32] Verbiest, T. ; Clays, K. ; Rodriguez, V. Second-Order Nonlinear Optical Characterization Techniques: An Introduction; CRC Press: Boca Raton, FL, 2009.

[33] Rodriguez V., Grondin J., Adamietz F., Danten Y. (2010). Local Structure in
Ionic Liquids Investigated by Hyper-Rayleigh Scattering. J. Phys. Chem. B.

[34] Castet F., Bogdan E., Plaquet A., Ducasse L., Champagne B., Rodriguez V. (2012). Reference
Molecules for Nonlinear Optics: A Joint Experimental
and Theoretical Investigation. J. Chem. Phys..

[35] van
Gisbergen S. J. A., Snijders J. G., Baerends E. J. (1998). Calculating Frequency-Dependent
Hyperpolarizabilities Using Time-Dependent Density Functional Theory. J. Chem. Phys..

[36] Helgaker T., Coriani S., Jørgensen P., Kristensen K., Olsen J., Ruud K. (2012). Recent Advances in
Wave Function-Based
Methods of Molecular-Property Calculations. Chem. Rev..

[37] Stratmann R. E., Scuseria G. E., Frisch M. J. (1998). An Efficient
Implementation of Time-Dependent
Density-Functional Theory for the Calculation of Excitation Energies
of Large Molecules. J. Chem. Phys..

[38] Franco L. R., Brandão I., Fonseca T. L., Georg H. C. (2016). Elucidating the
Structure of Merocyanine Dyes with the ASEC-FEG Method: Phenol Blue
in Solution. J. Chem. Phys..

[39] Stephens P.
J., Devlin F. J., Chabalowski C. F., Frisch M. J. (1994). Ab Initio Calculation
of Vibrational Absorption and Circular Dichroism Spectra Using Density
Functional Force Fields. J. Phys. Chem..

[40] Lu T., Chen F. (2012). Multiwfn: A Multifunctional Wavefunction Analyzer. J. Comput. Chem..

[41] Chaudhary J., Aarzoo, Roy R., Roy R. K. (2022). Screening
the Band Shape of Molecules by Optimal Tuning
of Range-Separated Hybrid Functional with TD-DFT: A Molecular Designing
Approach. J. Phys. Chem. A.

