# Supplementary material for: Solvent Polarity as a Selective Modulator of the First Hyperpolarizability in Para-Substituted Azo–Carbazole Dyes
Source: ACS Omega. 2026 Apr 29;11(18):26468–78. doi: 10.1021/acsomega.5c12352 (PMC13176964; doi:10.1021/acsomega.5c12352)
Supplement: Supplementary file 1 [file ao5c12352_si_001.pdf]

Supporting Information

Solvent Polarity as a Selective Modulator of the First Hyperpolarizability in Para-Substituted Azo-Carbazole Dyes

Murilo B. M. Ferreira, Herbert C. Georg, Marcos A. Castro, Tertius L. Fonseca*

Instituto de Física, Universidade Federal de Goiás, Goiânia-GO, 74690-900, Brazil.

Tables S1–S21 report the optimized ground-state bond lengths and bond-length alternation (BLA) parameters for all azo-carbazole derivatives investigated in this work, calculated in the gas phase and in different solvent environments using the PCM model. Tables S1–S7 correspond to CAM-B3LYP/6-311++G(d,p) results, Tables S8–S14 to ωB97X-D/6-311++G(d,p), and Tables S15–S21 to M06-2X/6-311++G(d,p). These data are provided to validate the robustness of the geometric trends discussed in the main text and to demonstrate that solvent effects induce only minor structural variations, while substituent effects dominate the BLA behavior.

Table S1: Bond Lengths and Bond-Length Alternation (BLA) Coordinates (in Å) Obtained for AmACzE in Gas Phase (GAS) and in Different Solvent Environments: Dichloromethane (DCM), Acetone (ACE), Methanol (MET), Dimethyl Sulfoxide (DMSO), and Water (WAT), for the ground state, calculated at the CAM-B3LYP/6-311++G(d,p) level.

	GAS	DCM	ACE	MET	DMSO	WAT
**H1-O2**	0.960	0.961	0.961	0.961	0.961	0.962
**C3-O2**	1.417	1.420	1.421	1.421	1.421	1.421
**C4-C3**	1.522	1.522	1.522	1.522	1.522	1.522
**C4-N5**	1.444	1.448	1.448	1.448	1.448	1.448
**C6-N5**	1.388	1.387	1.387	1.387	1.387	1.387
**C6-C7**	1.392	1.393	1.393	1.393	1.393	1.393
**C6-C11**	1.409	1.409	1.410	1.410	1.410	1.410
**C7-C8**	1.385	1.385	1.385	1.385	1.385	1.385
**C8-C9**	1.399	1.400	1.401	1.401	1.401	1.401
**C9-C10**	1.384	1.385	1.385	1.385	1.385	1.385
**C10-C11**	1.392	1.393	1.393	1.393	1.393	1.394
**C12-C11**	1.447	1.447	1.447	1.447	1.447	1.447
**C12-C17**	1.389	1.389	1.390	1.390	1.390	1.390
**C13-N5**	1.382	1.380	1.379	1.379	1.379	1.379
**C13-C12**	1.409	1.410	1.411	1.411	1.411	1.411
**C14-C13**	1.397	1.398	1.398	1.398	1.398	1.398
**C15-C14**	1.378	1.378	1.378	1.378	1.378	1.378
**C16-C15**	1.406	1.408	1.408	1.408	1.408	1.408
**C16-C17**	1.387	1.389	1.389	1.389	1.389	1.389
**C16-N18**	1.416	1.416	1.416	1.416	1.416	1.416
**N18-N19**	1.243	1.244	1.244	1.244	1.245	1.245
**C20-N19**	1.413	1.411	1.411	1.411	1.411	1.411
**C21-C20**	1.391	1.393	1.394	1.394	1.394	1.394
**C22-C21**	1.383	1.382	1.382	1.382	1.382	1.382
**C23-C22**	1.397	1.399	1.400	1.400	1.400	1.400
**C23-N41**	1.386	1.381	1.380	1.380	1.380	1.379
**C24-C23**	1.402	1.405	1.405	1.406	1.406	1.406
**C25-C20**	1.397	1.399	1.399	1.399	1.399	1.399
**C25-C24**	1.378	1.378	1.378	1.378	1.378	1.378
**H42-N41**	1.007	1.008	1.008	1.008	1.008	1.008
**H43-N41**	1.007	1.008	1.008	1.008	1.008	1.008
**BLA**	0.016	0.019	0.020	0.020	0.020	0.020

Table S2: Bond Lengths and Bond-Length Alternation (BLA) Coordinates (in Å) Obtained for MACzE in Gas Phase (GAS) and in Different Solvent Environments: Dichloromethane (DCM), Acetone (ACE), Methanol (MET), Dimethyl Sulfoxide (DMSO), and Water (WAT), for the ground state, calculated at the CAM-B3LYP/6-311++G(d,p) level.

	GAS	DCM	ACE	MET	DMSO	WAT
**H1-O2**	0.960	0.961	0.961	0.961	0.962	0.962
**C3-O2**	1.417	1.420	1.421	1.421	1.421	1.421
**C4-C3**	1.522	1.522	1.521	1.521	1.521	1.521
**C4-N5**	1.445	1.448	1.448	1.448	1.448	1.448
**C6-N5**	1.388	1.388	1.388	1.388	1.388	1.388
**C6-C7**	1.392	1.393	1.393	1.393	1.393	1.393
**C6-C11**	1.409	1.409	1.409	1.409	1.409	1.409
**C7-C8**	1.385	1.385	1.385	1.385	1.385	1.385
**C8-C9**	1.399	1.400	1.401	1.401	1.401	1.401
**C9-C10**	1.384	1.385	1.385	1.385	1.385	1.385
**C10-C11**	1.392	1.393	1.393	1.393	1.393	1.393
**C12-C11**	1.447	1.447	1.447	1.447	1.447	1.447
**C12-C17**	1.389	1.389	1.389	1.389	1.389	1.389
**C13-N5**	1.381	1.379	1.378	1.378	1.378	1.378
**C13-C12**	1.409	1.411	1.411	1.411	1.411	1.411
**C14-C13**	1.397	1.398	1.398	1.398	1.398	1.398
**C15-C14**	1.378	1.378	1.378	1.378	1.378	1.378
**C16-C15**	1.406	1.408	1.408	1.408	1.408	1.408
**C16-C17**	1.387	1.389	1.389	1.389	1.389	1.389
**C16-N18**	1.415	1.415	1.415	1.415	1.415	1.415
**N18-N19**	1.242	1.243	1.243	1.243	1.243	1.243
**C20-N19**	1.416	1.416	1.416	1.416	1.416	1.416
**C21-C20**	1.395	1.395	1.396	1.396	1.396	1.396
**C22-C21**	1.379	1.380	1.380	1.380	1.380	1.380
**C22-C23**	1.394	1.395	1.396	1.396	1.396	1.396
**C23-O41**	1.356	1.354	1.354	1.354	1.353	1.353
**C24-C23**	1.395	1.397	1.397	1.397	1.397	1.397
**C25-C20**	1.391	1.393	1.393	1.393	1.393	1.393
**C25-C24**	1.385	1.385	1.385	1.385	1.385	1.385
**C42-O41**	1.414	1.421	1.422	1.422	1.423	1.423
**BLA**	0.012	0.013	0.013	0.013	0.013	0.013

Table S3: Bond Lengths and Bond-Length Alternation (BLA) Coordinates (in Å) Obtained for HACzE in Gas Phase (GAS) and in Different Solvent Environments: Dichloromethane (DCM), Acetone (ACE), Methanol (MET), Dimethyl Sulfoxide (DMSO), and Water (WAT), for the ground state, calculated at the CAM-B3LYP/6-311++G(d,p) level.

	GAS	DCM	ACE	MET	DMSO	WAT
**H1-O2**	0.960	0.961	0.961	0.961	0.961	0.962
**C3-O2**	1.417	1.420	1.421	1.421	1.421	1.421
**C4-C3**	1.522	1.522	1.522	1.521	1.521	1.521
**C4-N5**	1.445	1.448	1.448	1.448	1.448	1.448
**C6-N5**	1.388	1.388	1.388	1.388	1.388	1.388
**C6-C7**	1.392	1.393	1.393	1.393	1.393	1.393
**C6-C11**	1.409	1.409	1.409	1.409	1.409	1.409
**C7-C8**	1.385	1.385	1.385	1.385	1.385	1.385
**C8-C9**	1.399	1.400	1.401	1.401	1.401	1.401
**C9-C10**	1.384	1.385	1.385	1.385	1.385	1.385
**C10-C11**	1.392	1.393	1.393	1.393	1.393	1.393
**C12-C11**	1.447	1.447	1.447	1.447	1.447	1.447
**C12-C17**	1.389	1.389	1.389	1.389	1.389	1.389
**C13-N5**	1.381	1.379	1.378	1.378	1.378	1.378
**C13-C12**	1.409	1.411	1.411	1.411	1.411	1.411
**C14-C13**	1.397	1.398	1.398	1.398	1.399	1.399
**C15-C14**	1.378	1.378	1.378	1.378	1.378	1.378
**C16-C15**	1.406	1.408	1.408	1.408	1.408	1.408
**C16-C17**	1.388	1.389	1.389	1.389	1.389	1.389
**C16-N18**	1.415	1.415	1.415	1.415	1.415	1.415
**N18-N19**	1.242	1.243	1.243	1.243	1.243	1.243
**C20-N19**	1.416	1.416	1.416	1.416	1.416	1.416
**C21-C20**	1.389	1.391	1.391	1.391	1.391	1.391
**C22-C21**	1.386	1.386	1.386	1.386	1.386	1.386
**C23-C22**	1.389	1.390	1.390	1.390	1.390	1.390
**C23-O41**	1.360	1.359	1.359	1.359	1.359	1.359
**C24-C23**	1.394	1.395	1.396	1.396	1.396	1.396
**C25-C20**	1.398	1.398	1.398	1.398	1.398	1.398
**C25-C24**	1.379	1.379	1.379	1.379	1.379	1.379
**H42-O41**	0.961	0.963	0.964	0.964	0.964	0.964
**BLA**	0.010	0.011	0.011	0.011	0.011	0.011

Table S4: Bond Lengths and Bond-Length Alternation (BLA) Coordinates (in Å) Obtained for FACzE in Gas Phase (GAS) and in Different Solvent Environments: Dichloromethane (DCM), Acetone (ACE), Methanol (MET), Dimethyl Sulfoxide (DMSO), and Water (WAT), for the ground state, calculated at the CAM-B3LYP/6-311++G(d,p) level.

	GAS	DCM	ACE	MET	DMSO	WAT
**H1-O2**	0.960	0.961	0.961	0.961	0.962	0.962
**C3-O2**	1.417	1.420	1.420	1.421	1.421	1.421
**C4-C3**	1.522	1.522	1.522	1.521	1.521	1.521
**C4-N5**	1.445	1.448	1.448	1.448	1.448	1.449
**C6-N5**	1.389	1.389	1.389	1.389	1.389	1.389
**C6-C7**	1.391	1.393	1.393	1.393	1.393	1.393
**C6-C11**	1.408	1.409	1.409	1.409	1.409	1.409
**C7-C8**	1.385	1.385	1.385	1.385	1.385	1.385
**C8-C9**	1.399	1.400	1.401	1.401	1.401	1.401
**C9-C10**	1.384	1.385	1.385	1.385	1.385	1.385
**C10-C11**	1.392	1.393	1.393	1.393	1.393	1.393
**C12-C11**	1.447	1.447	1.447	1.447	1.447	1.447
**C12-C17**	1.388	1.388	1.388	1.388	1.388	1.388
**C13-N5**	1.380	1.377	1.377	1.377	1.377	1.377
**C13-C12**	1.410	1.411	1.411	1.412	1.412	1.412
**C14-C13**	1.398	1.399	1.399	1.399	1.399	1.399
**C15-C14**	1.377	1.377	1.377	1.377	1.377	1.377
**C16-C15**	1.407	1.408	1.409	1.409	1.409	1.409
**C16-C17**	1.388	1.389	1.389	1.389	1.389	1.389
**C16-N18**	1.414	1.413	1.413	1.413	1.413	1.413
**N18-N19**	1.241	1.242	1.242	1.242	1.242	1.242
**C20-N19**	1.420	1.420	1.420	1.420	1.420	1.420
**C21-C20**	1.391	1.392	1.392	1.392	1.392	1.392
**C22-C21**	1.387	1.387	1.387	1.387	1.387	1.387
**C23-C22**	1.379	1.379	1.379	1.379	1.379	1.379
**C23-F41**	1.348	1.352	1.353	1.353	1.353	1.353
**C24-C23**	1.384	1.384	1.384	1.384	1.384	1.384
**C25-C20**	1.396	1.397	1.397	1.397	1.397	1.397
**C25-C24**	1.383	1.383	1.383	1.383	1.383	1.383
**BLA**	0.002	0.003	0.003	0.003	0.003	0.003

Table S5: Bond Lengths and Bond-Length Alternation (BLA) Coordinates (in Å) Obtained for AACzE in Gas Phase (GAS) and in Different Solvent Environments: Dichloromethane (DCM), Acetone (ACE), Methanol (MET), Dimethyl Sulfoxide (DMSO), and Water (WAT), for the ground state, calculated at the CAM-B3LYP/6-311++G(d,p) level.

	GAS	DCM	ACE	MET	DMSO	WAT
**H1-O2**	0.960	0.961	0.961	0.961	0.962	0.962
**C3-O2**	1.417	1.420	1.420	1.420	1.420	1.421
**C4-C3**	1.522	1.522	1.521	1.521	1.521	1.521
**C4-N5**	1.445	1.448	1.449	1.449	1.449	1.449
**C6-N5**	1.389	1.389	1.389	1.389	1.389	1.389
**C6-C7**	1.391	1.392	1.392	1.392	1.392	1.392
**C6-C11**	1.408	1.409	1.409	1.409	1.409	1.409
**C7-C8**	1.385	1.385	1.385	1.385	1.385	1.385
**C8-C9**	1.399	1.400	1.400	1.401	1.401	1.401
**C9-C10**	1.384	1.385	1.385	1.385	1.385	1.385
**C10-C11**	1.392	1.393	1.393	1.393	1.393	1.393
**C12-C11**	1.447	1.447	1.447	1.447	1.447	1.447
**C12-C17**	1.387	1.387	1.387	1.387	1.387	1.387
**C13-N5**	1.379	1.376	1.376	1.376	1.375	1.375
**C13-C12**	1.410	1.412	1.412	1.412	1.412	1.412
**C14-C13**	1.398	1.399	1.399	1.400	1.400	1.400
**C15-C14**	1.377	1.377	1.376	1.376	1.376	1.376
**C16-C15**	1.407	1.409	1.409	1.409	1.409	1.409
**C16-C17**	1.389	1.390	1.390	1.390	1.391	1.391
**C16-N18**	1.412	1.410	1.410	1.410	1.410	1.410
**N18-N19**	1.242	1.243	1.243	1.243	1.243	1.243
**C20-N19**	1.421	1.421	1.421	1.421	1.421	1.421
**C21-C20**	1.392	1.393	1.393	1.393	1.393	1.393
**C22-C21**	1.383	1.383	1.383	1.383	1.383	1.383
**C22-C23**	1.394	1.394	1.395	1.395	1.395	1.395
**C23-C42**	1.497	1.495	1.495	1.495	1.495	1.495
**C24-C23**	1.397	1.398	1.398	1.398	1.398	1.398
**C25-C20**	1.394	1.395	1.395	1.395	1.395	1.395
**C25-C24**	1.383	1.383	1.383	1.383	1.383	1.383
**O41-C42**	1.211	1.216	1.217	1.217	1.217	1.217
**C42-C43**	1.510	1.506	1.505	1.505	1.505	1.504
**BLA**	0.011	0.012	0.012	0.012	0.012	0.012

Table S6: Bond Lengths and Bond-Length Alternation (BLA) Coordinates (in Å) Obtained for CACzE in Gas Phase (GAS) and in Different Solvent Environments: Dichloromethane (DCM), Acetone (ACE), Methanol (MET), Dimethyl Sulfoxide (DMSO), and Water (WAT), for the ground state, calculated at the CAM-B3LYP/6-311++G(d,p) level.

	GAS	DCM	ACE	MET	DMSO	WAT
**H1-O2**	0.960	0.961	0.961	0.962	0.962	0.962
**C3-O2**	1.417	1.420	1.420	1.420	1.420	1.420
**C4-C3**	1.522	1.522	1.521	1.521	1.521	1.521
**C4-N5**	1.446	1.448	1.449	1.449	1.449	1.449
**C6-N5**	1.390	1.390	1.390	1.390	1.390	1.390
**C6-C7**	1.391	1.392	1.392	1.392	1.392	1.392
**C6-C11**	1.408	1.408	1.409	1.409	1.409	1.409
**C7-C8**	1.385	1.385	1.385	1.385	1.385	1.385
**C8-C9**	1.399	1.400	1.400	1.400	1.401	1.401
**C9-C10**	1.384	1.385	1.385	1.385	1.385	1.385
**C10-C11**	1.392	1.393	1.393	1.393	1.393	1.393
**C12-C11**	1.447	1.447	1.447	1.447	1.447	1.447
**C12-C17**	1.387	1.387	1.387	1.387	1.387	1.387
**C13-N5**	1.378	1.375	1.375	1.375	1.375	1.375
**C13-C12**	1.411	1.412	1.413	1.413	1.413	1.413
**C14-C13**	1.399	1.400	1.400	1.400	1.400	1.400
**C15-C14**	1.377	1.376	1.376	1.376	1.376	1.376
**C16-C15**	1.408	1.409	1.410	1.410	1.410	1.410
**C16-C17**	1.389	1.391	1.391	1.391	1.391	1.391
**C16-N18**	1.410	1.408	1.408	1.408	1.408	1.408
**N18-N19**	1.242	1.244	1.244	1.244	1.244	1.244
**C20-N19**	1.421	1.421	1.421	1.421	1.421	1.421
**C21-C20**	1.391	1.392	1.392	1.392	1.392	1.392
**C22-C21**	1.383	1.383	1.383	1.383	1.383	1.383
**C22-C23**	1.394	1.394	1.394	1.394	1.395	1.395
**C24-C23**	1.398	1.399	1.399	1.399	1.399	1.399
**C25-C20**	1.396	1.396	1.396	1.396	1.396	1.396
**C25-C24**	1.380	1.380	1.380	1.380	1.380	1.380
**C41-C23**	1.432	1.431	1.431	1.431	1.431	1.431
**C41-N42**	1.149	1.150	1.150	1.150	1.150	1.150
**BLA**	0.013	0.014	0.014	0.014	0.014	0.014

Table S7: Bond Lengths and Bond-Length Alternation (BLA) Coordinates (in Å) Obtained for NACzE in Gas Phase (GAS) and in Different Solvent Environments: Dichloromethane (DCM), Acetone (ACE), Methanol (MET), Dimethyl Sulfoxide (DMSO), and Water (WAT), for the ground state, calculated at the CAM-B3LYP/6-311++G(d,p) level.

	GAS	DCM	ACE	MET	DMSO	WAT
**H1-O2**	0.960	0.961	0.961	0.962	0.962	0.962
**C3-O2**	1.416	1.420	1.420	1.420	1.420	1.420
**C4-C3**	1.522	1.522	1.521	1.521	1.521	1.521
**C4-N5**	1.446	1.449	1.449	1.449	1.449	1.449
**C6-N5**	1.390	1.390	1.390	1.390	1.390	1.390
**C6-C7**	1.391	1.392	1.392	1.392	1.392	1.392
**C6-C11**	1.408	1.408	1.408	1.408	1.408	1.408
**C7-C8**	1.385	1.385	1.385	1.385	1.385	1.385
**C8-C9**	1.399	1.400	1.400	1.400	1.400	1.401
**C9-C10**	1.384	1.385	1.385	1.385	1.385	1.385
**C10-C11**	1.392	1.393	1.393	1.393	1.393	1.393
**C12-C11**	1.447	1.447	1.447	1.447	1.447	1.447
**C12-C17**	1.387	1.386	1.387	1.387	1.387	1.387
**C13-N5**	1.377	1.375	1.374	1.374	1.374	1.374
**C13-C12**	1.411	1.413	1.413	1.413	1.413	1.413
**C14-C13**	1.399	1.400	1.400	1.400	1.400	1.400
**C15-C14**	1.376	1.376	1.376	1.376	1.376	1.376
**C16-C15**	1.408	1.410	1.410	1.410	1.410	1.410
**C16-C17**	1.390	1.391	1.391	1.391	1.391	1.391
**C16-N18**	1.408	1.406	1.406	1.406	1.406	1.406
**N18-N19**	1.243	1.244	1.244	1.244	1.244	1.244
**C20-N19**	1.421	1.420	1.420	1.420	1.420	1.420
**C21-C20**	1.392	1.393	1.393	1.393	1.393	1.393
**C22-C21**	1.384	1.383	1.383	1.383	1.383	1.383
**C23-C22**	1.384	1.385	1.385	1.385	1.385	1.385
**C23-N41**	1.472	1.466	1.466	1.465	1.465	1.465
**C24-C23**	1.388	1.389	1.389	1.389	1.389	1.389
**C25-C20**	1.396	1.397	1.397	1.397	1.397	1.397
**C25-C24**	1.381	1.380	1.380	1.380	1.380	1.380
**N41-O42**	1.216	1.218	1.219	1.219	1.219	1.219
**N41-O43**	1.216	1.218	1.219	1.219	1.219	1.219
**BLA**	0.007	0.010	0.010	0.010	0.010	0.010

Table S8: Bond Lengths and Bond-Length Alternation (BLA) Coordinates (in Å) Obtained for AmACzE in Gas Phase (GAS) and in Different Solvent Environments: Dichloromethane (DCM), Acetone (ACE), Methanol (MET), Dimethyl Sulfoxide (DMSO), and Water (WAT), for the ground state, calculated at the wB97XD/6-311++G(d,p) level.

	GAS	DCM	ACE	MET	DMSO	WAT
**H1-O2**	0.957	0.958	0.959	0.959	0.959	0.959
**C3-O2**	1.414	1.416	1.417	1.417	1.417	1.417
**C4-C3**	1.523	1.523	1.523	1.523	1.523	1.523
**C4-N5**	1.443	1.446	1.446	1.446	1.446	1.447
**C6-N5**	1.386	1.386	1.386	1.386	1.386	1.386
**C6-C7**	1.393	1.395	1.395	1.395	1.395	1.395
**C6-C11**	1.409	1.410	1.410	1.410	1.410	1.410
**C7-C8**	1.387	1.387	1.387	1.387	1.387	1.387
**C8-C9**	1.401	1.402	1.403	1.403	1.403	1.403
**C9-C10**	1.386	1.387	1.387	1.387	1.387	1.387
**C10-C11**	1.393	1.395	1.395	1.395	1.395	1.395
**C12-C11**	1.447	1.447	1.447	1.447	1.447	1.447
**C12-C17**	1.390	1.391	1.391	1.391	1.391	1.391
**C13-N5**	1.381	1.379	1.378	1.378	1.378	1.378
**C13-C12**	1.409	1.411	1.411	1.411	1.411	1.411
**C14-C13**	1.398	1.399	1.399	1.399	1.399	1.399
**C15-C14**	1.380	1.380	1.380	1.380	1.380	1.380
**C16-C15**	1.408	1.410	1.410	1.410	1.410	1.410
**C16-C17**	1.389	1.390	1.390	1.390	1.390	1.390
**C16-N18**	1.417	1.418	1.417	1.417	1.417	1.417
**N18-N19**	1.244	1.245	1.245	1.246	1.246	1.246
**C20-N19**	1.414	1.413	1.413	1.412	1.412	1.412
**C21-C20**	1.393	1.395	1.395	1.395	1.395	1.395
**C22-C21**	1.385	1.384	1.384	1.384	1.384	1.384
**C23-C22**	1.399	1.401	1.402	1.402	1.402	1.402
**C23-N41**	1.387	1.382	1.381	1.381	1.381	1.381
**C24-C23**	1.404	1.407	1.407	1.407	1.407	1.408
**C25-C20**	1.398	1.400	1.401	1.401	1.401	1.401
**C25-C24**	1.380	1.380	1.380	1.380	1.380	1.380
**H42-N41**	1.007	1.008	1.008	1.008	1.008	1.008
**H43-N41**	1.007	1.008	1.008	1.008	1.008	1.008
**BLA**	0.016	0.019	0.019	0.019	0.019	0.020

Table S9: Bond Lengths and Bond-Length Alternation (BLA) Coordinates (in Å) Obtained for MACzE in Gas Phase (GAS) and in Different Solvent Environments: Dichloromethane (DCM), Acetone (ACE), Methanol (MET), Dimethyl Sulfoxide (DMSO), and Water (WAT), for the ground state, calculated at the wB97XD/6-311++G(d,p) level.

	GAS	DCM	ACE	MET	DMSO	WAT
**H1-O2**	0.957	0.959	0.959	0.959	0.959	0.959
**C3-O2**	1.414	1.416	1.417	1.417	1.417	1.417
**C4-C3**	1.523	1.523	1.523	1.523	1.523	1.523
**C4-N5**	1.443	1.446	1.446	1.447	1.447	1.447
**C6-N5**	1.387	1.386	1.386	1.386	1.386	1.386
**C6-C7**	1.393	1.394	1.395	1.395	1.395	1.395
**C6-C11**	1.409	1.410	1.410	1.410	1.410	1.410
**C7-C8**	1.387	1.387	1.387	1.387	1.387	1.387
**C8-C9**	1.401	1.402	1.403	1.403	1.403	1.403
**C9-C10**	1.386	1.387	1.387	1.387	1.387	1.387
**C10-C11**	1.393	1.395	1.395	1.395	1.395	1.395
**C12-C11**	1.447	1.447	1.447	1.447	1.447	1.447
**C12-C17**	1.390	1.391	1.391	1.391	1.391	1.391
**C13-N5**	1.380	1.377	1.377	1.377	1.377	1.377
**C13-C12**	1.410	1.411	1.411	1.412	1.412	1.412
**C14-C13**	1.399	1.400	1.400	1.400	1.400	1.400
**C15-C14**	1.380	1.379	1.379	1.379	1.379	1.379
**C16-C15**	1.408	1.410	1.410	1.410	1.410	1.410
**C16-C17**	1.389	1.390	1.391	1.391	1.391	1.391
**C16-N18**	1.417	1.416	1.416	1.416	1.416	1.416
**N18-N19**	1.243	1.244	1.244	1.244	1.244	1.244
**C20-N19**	1.417	1.417	1.417	1.417	1.417	1.417
**C21-C20**	1.396	1.397	1.397	1.397	1.397	1.397
**C22-C21**	1.381	1.382	1.382	1.382	1.382	1.382
**C22-C23**	1.397	1.398	1.398	1.398	1.398	1.398
**C23-O41**	1.353	1.351	1.351	1.351	1.351	1.351
**C24-C23**	1.398	1.399	1.399	1.399	1.399	1.399
**C25-C20**	1.393	1.395	1.395	1.395	1.395	1.395
**C25-C24**	1.387	1.387	1.387	1.387	1.387	1.387
**C42-O41**	1.412	1.419	1.420	1.420	1.420	1.420
**BLA**	0.012	0.013	0.013	0.013	0.013	0.013

Table S10: Bond Lengths and Bond-Length Alternation (BLA) Coordinates (in Å) Obtained for HACzE in Gas Phase (GAS) and in Different Solvent Environments: Dichloromethane (DCM), Acetone (ACE), Methanol (MET), Dimethyl Sulfoxide (DMSO), and Water (WAT), for the ground state, calculated at the wB97XD/6-311++G(d,p) level.

	GAS	DCM	ACE	MET	DMSO	WAT
**H1-O2**	0.957	0.958	0.959	0.959	0.959	0.959
**C3-O2**	1.414	1.416	1.417	1.417	1.417	1.417
**C4-C3**	1.523	1.523	1.523	1.523	1.523	1.523
**C4-N5**	1.443	1.446	1.447	1.447	1.447	1.447
**C6-N5**	1.387	1.387	1.386	1.386	1.386	1.386
**C6-C7**	1.393	1.394	1.395	1.395	1.395	1.395
**C6-C11**	1.409	1.410	1.410	1.410	1.410	1.410
**C7-C8**	1.387	1.387	1.387	1.387	1.387	1.387
**C8-C9**	1.401	1.402	1.403	1.403	1.403	1.403
**C9-C10**	1.386	1.386	1.387	1.387	1.387	1.387
**C10-C11**	1.393	1.395	1.395	1.395	1.395	1.395
**C12-C11**	1.447	1.446	1.446	1.446	1.446	1.446
**C12-C17**	1.390	1.390	1.391	1.391	1.391	1.391
**C13-N5**	1.380	1.377	1.377	1.377	1.377	1.377
**C13-C12**	1.410	1.411	1.412	1.412	1.412	1.412
**C14-C13**	1.399	1.400	1.400	1.400	1.400	1.400
**C15-C14**	1.380	1.379	1.379	1.379	1.379	1.379
**C16-C15**	1.408	1.410	1.410	1.410	1.410	1.410
**C16-C17**	1.389	1.390	1.391	1.391	1.391	1.391
**C16-N18**	1.417	1.416	1.416	1.416	1.416	1.416
**N18-N19**	1.243	1.244	1.244	1.244	1.244	1.244
**C20-N19**	1.417	1.417	1.417	1.417	1.417	1.417
**C21-C20**	1.391	1.393	1.393	1.393	1.393	1.393
**C22-C21**	1.388	1.388	1.388	1.388	1.388	1.388
**C23-C22**	1.392	1.393	1.393	1.393	1.393	1.393
**C23-O41**	1.358	1.356	1.356	1.355	1.355	1.355
**C24-C23**	1.397	1.398	1.398	1.398	1.398	1.398
**C25-C20**	1.399	1.400	1.400	1.400	1.400	1.400
**C25-C24**	1.381	1.381	1.381	1.381	1.381	1.381
**H42-O41**	0.959	0.961	0.961	0.961	0.961	0.961
**BLA**	0.010	0.012	0.012	0.012	0.012	0.012

Table S11: Bond Lengths and Bond-Length Alternation (BLA) Coordinates (in Å) Obtained for FACzE in Gas Phase (GAS) and in Different Solvent Environments: Dichloromethane (DCM), Acetone (ACE), Methanol (MET), Dimethyl Sulfoxide (DMSO), and Water (WAT), for the ground state, calculated at the wB97XD/6-311++G(d,p) level.

	GAS	DCM	ACE	MET	DMSO	WAT
**H1-O2**	0.957	0.959	0.959	0.959	0.959	0.959
**C3-O2**	1.413	1.416	1.416	1.417	1.417	1.417
**C4-C3**	1.523	1.523	1.523	1.523	1.523	1.523
**C4-N5**	1.444	1.446	1.447	1.447	1.447	1.447
**C6-N5**	1.387	1.387	1.387	1.387	1.387	1.387
**C6-C7**	1.393	1.394	1.394	1.394	1.394	1.394
**C6-C11**	1.409	1.410	1.410	1.410	1.410	1.410
**C7-C8**	1.387	1.387	1.387	1.387	1.387	1.387
**C8-C9**	1.401	1.402	1.403	1.403	1.403	1.403
**C9-C10**	1.386	1.386	1.386	1.387	1.387	1.387
**C10-C11**	1.393	1.395	1.395	1.395	1.395	1.395
**C12-C11**	1.446	1.446	1.446	1.446	1.446	1.446
**C12-C17**	1.389	1.390	1.390	1.390	1.390	1.390
**C13-N5**	1.379	1.376	1.376	1.376	1.376	1.376
**C13-C12**	1.410	1.412	1.412	1.412	1.412	1.412
**C14-C13**	1.399	1.400	1.400	1.400	1.400	1.400
**C15-C14**	1.379	1.379	1.379	1.379	1.379	1.379
**C16-C15**	1.408	1.410	1.410	1.410	1.410	1.410
**C16-C17**	1.389	1.391	1.391	1.391	1.391	1.391
**C16-N18**	1.415	1.414	1.414	1.414	1.414	1.414
**N18-N19**	1.242	1.243	1.243	1.243	1.243	1.244
**C20-N19**	1.421	1.421	1.421	1.421	1.421	1.421
**C21-C20**	1.392	1.393	1.393	1.393	1.393	1.393
**C22-C21**	1.388	1.389	1.389	1.389	1.389	1.389
**C23-C22**	1.382	1.382	1.382	1.382	1.382	1.382
**C23-F41**	1.345	1.349	1.349	1.349	1.349	1.349
**C24-C23**	1.387	1.387	1.387	1.387	1.387	1.387
**C25-C20**	1.397	1.398	1.398	1.398	1.398	1.398
**C25-C24**	1.384	1.385	1.385	1.385	1.385	1.385
**BLA**	0.004	0.003	0.003	0.003	0.003	0.003

Table S12: Bond Lengths and Bond-Length Alternation (BLA) Coordinates (in Å) Obtained for AACzE in Gas Phase (GAS) and in Different Solvent Environments: Dichloromethane (DCM), Acetone (ACE), Methanol (MET), Dimethyl Sulfoxide (DMSO), and Water (WAT), for the ground state, calculated at the wB97XD/6-311++G(d,p) level.

	GAS	DCM	ACE	MET	DMSO	WAT
**H1-O2**	0.957	0.959	0.959	0.959	0.959	0.959
**C3-O2**	1.413	1.416	1.416	1.416	1.416	1.417
**C4-C3**	1.523	1.523	1.523	1.523	1.523	1.523
**C4-N5**	1.444	1.447	1.447	1.447	1.447	1.447
**C6-N5**	1.388	1.388	1.388	1.388	1.388	1.388
**C6-C7**	1.393	1.394	1.394	1.394	1.394	1.394
**C6-C11**	1.409	1.409	1.409	1.409	1.409	1.409
**C7-C8**	1.387	1.387	1.387	1.387	1.387	1.387
**C8-C9**	1.401	1.402	1.402	1.403	1.403	1.403
**C9-C10**	1.386	1.386	1.386	1.387	1.387	1.387
**C10-C11**	1.393	1.395	1.395	1.395	1.395	1.395
**C12-C11**	1.447	1.447	1.447	1.447	1.447	1.447
**C12-C17**	1.389	1.389	1.389	1.389	1.389	1.389
**C13-N5**	1.378	1.375	1.375	1.375	1.375	1.375
**C13-C12**	1.411	1.413	1.413	1.413	1.413	1.413
**C14-C13**	1.399	1.401	1.401	1.401	1.401	1.401
**C15-C14**	1.379	1.378	1.378	1.378	1.378	1.378
**C16-C15**	1.409	1.411	1.411	1.411	1.411	1.411
**C16-C17**	1.390	1.392	1.392	1.392	1.392	1.392
**C16-N18**	1.413	1.411	1.411	1.411	1.411	1.411
**N18-N19**	1.243	1.244	1.244	1.244	1.244	1.244
**C20-N19**	1.423	1.423	1.423	1.423	1.423	1.423
**C21-C20**	1.394	1.394	1.395	1.395	1.395	1.395
**C22-C21**	1.385	1.385	1.385	1.385	1.385	1.385
**C22-C23**	1.395	1.396	1.396	1.396	1.396	1.396
**C23-C42**	1.500	1.499	1.498	1.498	1.498	1.498
**C24-C23**	1.399	1.399	1.399	1.399	1.399	1.399
**C25-C20**	1.396	1.396	1.396	1.396	1.397	1.396
**C25-C24**	1.384	1.385	1.385	1.385	1.385	1.385
**O41-C42**	1.211	1.216	1.216	1.217	1.217	1.217
**C42-C43**	1.512	1.508	1.507	1.507	1.507	1.506
**BLA**	0.012	0.011	0.011	0.011	0.012	0.011

Table S13: Bond Lengths and Bond-Length Alternation (BLA) Coordinates (in Å) Obtained for CACzE in Gas Phase (GAS) and in Different Solvent Environments: Dichloromethane (DCM), Acetone (ACE), Methanol (MET), Dimethyl Sulfoxide (DMSO), and Water (WAT), for the ground state, calculated at the wB97XD/6-311++G(d,p) level.

	GAS	DCM	ACE	MET	DMSO	WAT
**H1-O2**	0.957	0.959	0.959	0.959	0.959	0.959
**C3-O2**	1.413	1.416	1.416	1.416	1.416	1.416
**C4-C3**	1.523	1.523	1.523	1.523	1.523	1.523
**C4-N5**	1.444	1.447	1.447	1.447	1.447	1.447
**C6-N5**	1.388	1.388	1.388	1.388	1.388	1.388
**C6-C7**	1.393	1.394	1.394	1.394	1.394	1.394
**C6-C11**	1.408	1.409	1.409	1.409	1.409	1.409
**C7-C8**	1.386	1.387	1.387	1.387	1.387	1.387
**C8-C9**	1.401	1.402	1.402	1.402	1.403	1.403
**C9-C10**	1.386	1.386	1.386	1.386	1.387	1.387
**C10-C11**	1.393	1.395	1.395	1.395	1.395	1.395
**C12-C11**	1.447	1.447	1.447	1.447	1.447	1.447
**C12-C17**	1.388	1.388	1.389	1.389	1.389	1.389
**C13-N5**	1.377	1.374	1.374	1.374	1.374	1.374
**C13-C12**	1.411	1.413	1.413	1.413	1.413	1.413
**C14-C13**	1.400	1.401	1.401	1.401	1.401	1.401
**C15-C14**	1.378	1.378	1.378	1.378	1.378	1.378
**C16-C15**	1.409	1.411	1.411	1.411	1.411	1.411
**C16-C17**	1.391	1.392	1.392	1.392	1.392	1.392
**C16-N18**	1.411	1.409	1.409	1.409	1.409	1.409
**N18-N19**	1.244	1.244	1.245	1.245	1.245	1.245
**C20-N19**	1.423	1.423	1.423	1.423	1.423	1.423
**C21-C20**	1.393	1.393	1.394	1.394	1.394	1.394
**C22-C21**	1.385	1.385	1.385	1.385	1.385	1.385
**C22-C23**	1.396	1.396	1.396	1.396	1.396	1.396
**C24-C23**	1.400	1.400	1.400	1.400	1.400	1.400
**C25-C20**	1.397	1.398	1.398	1.398	1.398	1.398
**C25-C24**	1.382	1.382	1.382	1.382	1.382	1.382
**C41-C23**	1.434	1.433	1.433	1.433	1.433	1.433
**C41-N42**	1.152	1.152	1.152	1.152	1.153	1.153
**BLA**	0.013	0.013	0.013	0.013	0.013	0.013

Table S14: Bond Lengths and Bond-Length Alternation (BLA) Coordinates (in Å) Obtained for NACzE in Gas Phase (GAS) and in Different Solvent Environments: Dichloromethane (DCM), Acetone (ACE), Methanol (MET), Dimethyl Sulfoxide (DMSO), and Water (WAT), for the ground state, calculated at the wB97XD/6-311++G(d,p) level.

	GAS	DCM	ACE	MET	DMSO	WAT
**H1-O2**	0.957	0.959	0.959	0.959	0.959	0.959
**C3-O2**	1.413	1.416	1.416	1.416	1.416	1.416
**C4-C3**	1.523	1.523	1.523	1.523	1.523	1.523
**C4-N5**	1.444	1.447	1.447	1.448	1.448	1.448
**C6-N5**	1.389	1.388	1.388	1.388	1.388	1.388
**C6-C7**	1.393	1.394	1.394	1.394	1.394	1.394
**C6-C11**	1.408	1.409	1.409	1.409	1.409	1.409
**C7-C8**	1.386	1.387	1.387	1.387	1.387	1.387
**C8-C9**	1.401	1.402	1.402	1.402	1.402	1.403
**C9-C10**	1.386	1.386	1.386	1.386	1.386	1.387
**C10-C11**	1.393	1.395	1.395	1.395	1.395	1.395
**C12-C11**	1.447	1.447	1.447	1.447	1.447	1.447
**C12-C17**	1.388	1.388	1.388	1.388	1.388	1.388
**C13-N5**	1.376	1.374	1.373	1.373	1.373	1.373
**C13-C12**	1.412	1.413	1.413	1.413	1.413	1.413
**C14-C13**	1.400	1.401	1.401	1.401	1.401	1.402
**C15-C14**	1.378	1.377	1.377	1.377	1.377	1.377
**C16-C15**	1.409	1.411	1.411	1.411	1.411	1.412
**C16-C17**	1.391	1.393	1.393	1.393	1.393	1.393
**C16-N18**	1.410	1.408	1.409	1.409	1.409	1.409
**N18-N19**	1.244	1.245	1.245	1.245	1.245	1.245
**C20-N19**	1.423	1.422	1.421	1.421	1.421	1.421
**C21-C20**	1.393	1.394	1.394	1.394	1.394	1.394
**C22-C21**	1.386	1.385	1.385	1.385	1.385	1.385
**C23-C22**	1.386	1.387	1.387	1.387	1.387	1.387
**C23-N41**	1.474	1.468	1.467	1.467	1.467	1.467
**C24-C23**	1.390	1.391	1.390	1.390	1.390	1.391
**C25-C20**	1.398	1.398	1.398	1.398	1.398	1.398
**C25-C24**	1.383	1.382	1.383	1.383	1.383	1.383
**N41-O42**	1.215	1.218	1.218	1.218	1.218	1.218
**N41-O43**	1.215	1.218	1.218	1.218	1.218	1.218
**BLA**	0.007	0.009	0.008	0.008	0.008	0.008

Table S15: Bond Lengths and Bond-Length Alternation (BLA) Coordinates (in Å) Obtained for AmACzE in Gas Phase (GAS) and in Different Solvent Environments: Dichloromethane (DCM), Acetone (ACE), Methanol (MET), Dimethyl Sulfoxide (DMSO), and Water (WAT), for the ground state, calculated at the M06-2X/6-311++G(d,p) level.

	GAS	DCM	ACE	MET	DMSO	WAT
**H1-O2**	0.959	0.961	0.961	0.961	0.961	0.961
**C3-O2**	1.414	1.417	1.417	1.417	1.417	1.417
**C4-C3**	1.523	1.523	1.523	1.523	1.523	1.523
**C4-N5**	1.445	1.448	1.448	1.448	1.449	1.449
**C6-N5**	1.387	1.386	1.386	1.386	1.386	1.386
**C6-C7**	1.395	1.396	1.396	1.396	1.396	1.396
**C6-C11**	1.412	1.413	1.413	1.413	1.413	1.413
**C7-C8**	1.387	1.388	1.388	1.388	1.388	1.388
**C8-C9**	1.402	1.404	1.404	1.404	1.404	1.404
**C9-C10**	1.387	1.387	1.387	1.387	1.387	1.387
**C10-C11**	1.395	1.396	1.396	1.397	1.397	1.397
**C12-C11**	1.448	1.448	1.448	1.448	1.448	1.448
**C12-C17**	1.392	1.393	1.393	1.393	1.393	1.393
**C13-N5**	1.381	1.379	1.378	1.378	1.378	1.378
**C13-C12**	1.412	1.414	1.414	1.414	1.414	1.414
**C14-C13**	1.400	1.401	1.401	1.401	1.401	1.401
**C15-C14**	1.381	1.381	1.381	1.381	1.381	1.381
**C16-C15**	1.410	1.412	1.412	1.412	1.412	1.412
**C16-C17**	1.390	1.391	1.391	1.391	1.391	1.391
**C16-N18**	1.419	1.419	1.419	1.419	1.419	1.419
**N18-N19**	1.242	1.244	1.244	1.244	1.244	1.244
**C20-N19**	1.417	1.415	1.415	1.415	1.415	1.415
**C21-C20**	1.394	1.396	1.396	1.396	1.396	1.396
**C22-C21**	1.385	1.385	1.385	1.385	1.385	1.385
**C23-C22**	1.399	1.402	1.402	1.403	1.403	1.403
**C23-N41**	1.389	1.383	1.382	1.382	1.382	1.381
**C24-C23**	1.405	1.408	1.408	1.409	1.409	1.409
**C25-C20**	1.400	1.401	1.402	1.402	1.402	1.402
**C25-C24**	1.381	1.381	1.381	1.381	1.381	1.381
**H42-N41**	1.008	1.009	1.010	1.010	1.010	1.010
**H43-N41**	1.008	1.009	1.010	1.010	1.010	1.010
**BLA**	0.016	0.019	0.019	0.019	0.019	0.019

Table S16: Bond Lengths and Bond-Length Alternation (BLA) Coordinates (in Å) Obtained for MACzE in Gas Phase (GAS) and in Different Solvent Environments: Dichloromethane (DCM), Acetone (ACE), Methanol (MET), Dimethyl Sulfoxide (DMSO), and Water (WAT), for the ground state, calculated at the M06-2X/6-311++G(d,p) level.

	GAS	DCM	ACE	MET	DMSO	WAT
**H1-O2**	0.959	0.961	0.961	0.961	0.961	0.961
**C3-O2**	1.414	1.416	1.417	1.417	1.417	1.417
**C4-C3**	1.523	1.523	1.523	1.523	1.523	1.523
**C4-N5**	1.445	1.448	1.449	1.449	1.449	1.449
**C6-N5**	1.387	1.387	1.387	1.387	1.387	1.387
**C6-C7**	1.395	1.396	1.396	1.396	1.396	1.396
**C6-C11**	1.412	1.413	1.413	1.413	1.413	1.413
**C7-C8**	1.387	1.388	1.388	1.388	1.388	1.388
**C8-C9**	1.402	1.404	1.404	1.404	1.404	1.404
**C9-C10**	1.387	1.387	1.387	1.387	1.387	1.387
**C10-C11**	1.395	1.396	1.397	1.397	1.397	1.397
**C12-C11**	1.448	1.448	1.448	1.448	1.448	1.448
**C12-C17**	1.392	1.392	1.392	1.392	1.392	1.392
**C13-N5**	1.380	1.377	1.377	1.377	1.377	1.377
**C13-C12**	1.413	1.414	1.415	1.415	1.415	1.415
**C14-C13**	1.400	1.401	1.402	1.402	1.402	1.402
**C15-C14**	1.381	1.380	1.380	1.380	1.380	1.380
**C16-C15**	1.410	1.412	1.412	1.412	1.412	1.412
**C16-C17**	1.390	1.391	1.391	1.391	1.391	1.391
**C16-N18**	1.419	1.419	1.419	1.419	1.419	1.419
**N18-N19**	1.242	1.243	1.243	1.243	1.243	1.243
**C20-N19**	1.420	1.420	1.420	1.420	1.420	1.420
**C21-C20**	1.397	1.398	1.398	1.398	1.398	1.398
**C22-C21**	1.382	1.383	1.383	1.383	1.383	1.383
**C22-C23**	1.397	1.399	1.399	1.399	1.399	1.399
**C23-O41**	1.355	1.353	1.353	1.353	1.353	1.353
**C24-C23**	1.399	1.400	1.400	1.400	1.400	1.400
**C25-C20**	1.394	1.395	1.395	1.395	1.396	1.396
**C25-C24**	1.388	1.388	1.388	1.388	1.388	1.388
**C42-O41**	1.413	1.420	1.421	1.421	1.421	1.421
**BLA**	0.012	0.013	0.013	0.013	0.013	0.013

Table S17: Bond Lengths and Bond-Length Alternation (BLA) Coordinates (in Å) Obtained for HACzE in Gas Phase (GAS) and in Different Solvent Environments: Dichloromethane (DCM), Acetone (ACE), Methanol (MET), Dimethyl Sulfoxide (DMSO), and Water (WAT), for the ground state, calculated at the M06-2X/6-311++G(d,p) level.

	GAS	DCM	ACE	MET	DMSO	WAT
**H1-O2**	0.959	0.961	0.961	0.961	0.961	0.961
**C3-O2**	1.414	1.417	1.417	1.417	1.417	1.417
**C4-C3**	1.523	1.523	1.523	1.523	1.523	1.523
**C4-N5**	1.445	1.448	1.449	1.449	1.449	1.449
**C6-N5**	1.387	1.387	1.387	1.387	1.387	1.387
**C6-C7**	1.395	1.396	1.396	1.396	1.396	1.396
**C6-C11**	1.412	1.413	1.413	1.413	1.413	1.413
**C7-C8**	1.387	1.388	1.388	1.388	1.388	1.388
**C8-C9**	1.402	1.404	1.404	1.404	1.404	1.404
**C9-C10**	1.387	1.387	1.387	1.387	1.387	1.387
**C10-C11**	1.395	1.396	1.396	1.396	1.397	1.397
**C12-C11**	1.448	1.448	1.448	1.448	1.448	1.448
**C12-C17**	1.391	1.392	1.392	1.392	1.392	1.392
**C13-N5**	1.380	1.378	1.377	1.377	1.377	1.377
**C13-C12**	1.413	1.414	1.415	1.415	1.415	1.415
**C14-C13**	1.400	1.402	1.402	1.402	1.402	1.402
**C15-C14**	1.381	1.380	1.380	1.380	1.380	1.380
**C16-C15**	1.410	1.412	1.412	1.412	1.412	1.412
**C16-C17**	1.390	1.391	1.391	1.391	1.391	1.391
**C16-N18**	1.419	1.419	1.419	1.419	1.419	1.419
**N18-N19**	1.242	1.243	1.243	1.243	1.243	1.243
**C20-N19**	1.420	1.420	1.420	1.420	1.420	1.420
**C21-C20**	1.391	1.393	1.393	1.393	1.393	1.393
**C22-C21**	1.389	1.389	1.389	1.389	1.389	1.389
**C23-C22**	1.392	1.393	1.393	1.393	1.393	1.393
**C23-O41**	1.359	1.357	1.357	1.357	1.357	1.357
**C24-C23**	1.398	1.399	1.399	1.399	1.399	1.399
**C25-C20**	1.401	1.401	1.401	1.401	1.401	1.401
**C25-C24**	1.381	1.382	1.382	1.382	1.382	1.382
**H42-O41**	0.961	0.963	0.963	0.963	0.964	0.964
**BLA**	0.010	0.011	0.011	0.011	0.011	0.011

Table S18: Bond Lengths and Bond-Length Alternation (BLA) Coordinates (in Å) Obtained for FACzE in Gas Phase (GAS) and in Different Solvent Environments: Dichloromethane (DCM), Acetone (ACE), Methanol (MET), Dimethyl Sulfoxide (DMSO), and Water (WAT), for the ground state, calculated at the M06-2X/6-311++G(d,p) level.

	GAS	DCM	ACE	MET	DMSO	WAT
**H1-O2**	0.959	0.961	0.961	0.961	0.961	0.961
**C3-O2**	1.414	1.416	1.417	1.417	1.417	1.417
**C4-C3**	1.523	1.523	1.523	1.523	1.523	1.523
**C4-N5**	1.445	1.448	1.449	1.449	1.449	1.449
**C6-N5**	1.387	1.387	1.387	1.387	1.387	1.387
**C6-C7**	1.395	1.396	1.396	1.396	1.396	1.396
**C6-C11**	1.412	1.412	1.413	1.413	1.413	1.413
**C7-C8**	1.387	1.388	1.388	1.388	1.388	1.388
**C8-C9**	1.402	1.404	1.404	1.404	1.404	1.404
**C9-C10**	1.387	1.387	1.387	1.387	1.387	1.387
**C10-C11**	1.395	1.396	1.396	1.396	1.397	1.397
**C12-C11**	1.448	1.448	1.448	1.448	1.448	1.448
**C12-C17**	1.391	1.391	1.391	1.391	1.391	1.391
**C13-N5**	1.379	1.377	1.376	1.376	1.376	1.376
**C13-C12**	1.413	1.415	1.415	1.415	1.415	1.415
**C14-C13**	1.401	1.402	1.402	1.402	1.402	1.402
**C15-C14**	1.380	1.380	1.380	1.380	1.380	1.380
**C16-C15**	1.410	1.412	1.412	1.412	1.412	1.412
**C16-C17**	1.390	1.391	1.391	1.391	1.391	1.391
**C16-N18**	1.418	1.417	1.417	1.417	1.417	1.417
**N18-N19**	1.241	1.242	1.242	1.242	1.242	1.242
**C20-N19**	1.423	1.424	1.424	1.424	1.424	1.424
**C21-C20**	1.393	1.394	1.394	1.394	1.394	1.394
**C22-C21**	1.389	1.390	1.390	1.390	1.390	1.390
**C23-C22**	1.383	1.382	1.382	1.382	1.382	1.382
**C23-F41**	1.343	1.347	1.347	1.347	1.347	1.347
**C24-C23**	1.388	1.388	1.388	1.388	1.388	1.388
**C25-C20**	1.399	1.399	1.399	1.399	1.399	1.399
**C25-C24**	1.385	1.385	1.386	1.386	1.386	1.386
**BLA**	0.004	0.003	0.003	0.003	0.003	0.003

Table S19: Bond Lengths and Bond-Length Alternation (BLA) Coordinates (in Å) Obtained for AACzE in Gas Phase (GAS) and in Different Solvent Environments: Dichloromethane (DCM), Acetone (ACE), Methanol (MET), Dimethyl Sulfoxide (DMSO), and Water (WAT), for the ground state, calculated at the M06-2X/6-311++G(d,p) level.

	GAS	DCM	ACE	MET	DMSO	WAT
**H1-O2**	0.959	0.961	0.961	0.961	0.961	0.961
**C3-O2**	1.414	1.416	1.417	1.417	1.417	1.417
**C4-C3**	1.523	1.523	1.523	1.523	1.523	1.523
**C4-N5**	1.446	1.449	1.449	1.449	1.449	1.449
**C6-N5**	1.388	1.388	1.388	1.388	1.388	1.388
**C6-C7**	1.395	1.395	1.396	1.396	1.396	1.396
**C6-C11**	1.411	1.412	1.412	1.412	1.412	1.412
**C7-C8**	1.387	1.388	1.388	1.388	1.388	1.388
**C8-C9**	1.402	1.404	1.404	1.404	1.404	1.404
**C9-C10**	1.387	1.387	1.387	1.387	1.387	1.387
**C10-C11**	1.395	1.396	1.396	1.396	1.396	1.397
**C12-C11**	1.448	1.448	1.448	1.448	1.448	1.448
**C12-C17**	1.391	1.391	1.391	1.391	1.391	1.391
**C13-N5**	1.378	1.375	1.375	1.375	1.375	1.375
**C13-C12**	1.414	1.415	1.416	1.416	1.416	1.416
**C14-C13**	1.401	1.403	1.403	1.403	1.403	1.403
**C15-C14**	1.379	1.379	1.379	1.379	1.379	1.379
**C16-C15**	1.411	1.412	1.413	1.413	1.413	1.413
**C16-C17**	1.391	1.392	1.392	1.392	1.392	1.392
**C16-N18**	1.416	1.414	1.414	1.414	1.414	1.414
**N18-N19**	1.242	1.243	1.243	1.243	1.243	1.243
**C20-N19**	1.425	1.425	1.425	1.425	1.425	1.425
**C21-C20**	1.395	1.396	1.396	1.396	1.396	1.396
**C22-C21**	1.385	1.386	1.386	1.386	1.386	1.386
**C22-C23**	1.396	1.397	1.397	1.397	1.397	1.397
**C23-C42**	1.501	1.500	1.500	1.500	1.500	1.500
**C24-C23**	1.399	1.400	1.400	1.400	1.400	1.400
**C25-C20**	1.397	1.398	1.398	1.398	1.398	1.398
**C25-C24**	1.386	1.386	1.386	1.386	1.386	1.386
**O41-C42**	1.209	1.214	1.214	1.214	1.214	1.214
**C42-C43**	1.512	1.508	1.507	1.507	1.507	1.507
**BLA**	0.011	0.012	0.012	0.012	0.012	0.012

Table S20: Bond Lengths and Bond-Length Alternation (BLA) Coordinates (in Å) Obtained for CACzE in Gas Phase (GAS) and in Different Solvent Environments: Dichloromethane (DCM), Acetone (ACE), Methanol (MET), Dimethyl Sulfoxide (DMSO), and Water (WAT), for the ground state, calculated at the M06-2X/6-311++G(d,p) level.

	GAS	DCM	ACE	MET	DMSO	WAT
**H1-O2**	0.959	0.961	0.961	0.961	0.961	0.961
**C3-O2**	1.413	1.416	1.416	1.417	1.417	1.417
**C4-C3**	1.523	1.523	1.523	1.523	1.523	1.523
**C4-N5**	1.446	1.449	1.449	1.449	1.450	1.450
**C6-N5**	1.389	1.388	1.388	1.388	1.388	1.388
**C6-C7**	1.395	1.395	1.395	1.396	1.396	1.396
**C6-C11**	1.411	1.412	1.412	1.412	1.412	1.412
**C7-C8**	1.387	1.388	1.388	1.388	1.388	1.388
**C8-C9**	1.402	1.404	1.404	1.404	1.404	1.404
**C9-C10**	1.386	1.387	1.387	1.387	1.387	1.387
**C10-C11**	1.395	1.396	1.396	1.396	1.396	1.396
**C12-C11**	1.448	1.448	1.448	1.448	1.448	1.448
**C12-C17**	1.390	1.390	1.390	1.390	1.390	1.390
**C13-N5**	1.377	1.374	1.374	1.374	1.374	1.374
**C13-C12**	1.414	1.416	1.416	1.416	1.416	1.416
**C14-C13**	1.402	1.403	1.403	1.403	1.403	1.403
**C15-C14**	1.379	1.379	1.379	1.379	1.379	1.379
**C16-C15**	1.411	1.413	1.413	1.413	1.413	1.413
**C16-C17**	1.391	1.393	1.393	1.393	1.393	1.393
**C16-N18**	1.414	1.412	1.412	1.412	1.412	1.412
**N18-N19**	1.242	1.243	1.243	1.243	1.243	1.243
**C20-N19**	1.424	1.425	1.425	1.425	1.425	1.425
**C21-C20**	1.394	1.394	1.395	1.395	1.395	1.395
**C22-C21**	1.386	1.387	1.387	1.387	1.387	1.387
**C22-C23**	1.396	1.396	1.397	1.397	1.397	1.397
**C24-C23**	1.401	1.401	1.401	1.401	1.401	1.401
**C25-C20**	1.399	1.399	1.399	1.399	1.399	1.399
**C25-C24**	1.383	1.383	1.383	1.383	1.383	1.383
**C41-C23**	1.436	1.436	1.436	1.436	1.436	1.436
**C41-N42**	1.151	1.151	1.151	1.151	1.151	1.151
**BLA**	0.013	0.012	0.013	0.013	0.013	0.013

Table S21: Bond Lengths and Bond-Length Alternation (BLA) Coordinates (in Å) Obtained for NACzE in Gas Phase (GAS) and in Different Solvent Environments: Dichloromethane (DCM), Acetone (ACE), Methanol (MET), Dimethyl Sulfoxide (DMSO), and Water (WAT), for the ground state, calculated at the M06-2X/6-311++G(d,p) level.

	GAS	DCM	ACE	MET	DMSO	WAT
**H1-O2**	0.959	0.961	0.961	0.961	0.961	0.961
**C3-O2**	1.413	1.416	1.416	1.417	1.417	1.417
**C4-C3**	1.523	1.523	1.523	1.523	1.523	1.523
**C4-N5**	1.446	1.449	1.450	1.450	1.450	1.450
**C6-N5**	1.389	1.389	1.389	1.389	1.389	1.389
**C6-C7**	1.394	1.395	1.395	1.395	1.395	1.395
**C6-C11**	1.411	1.412	1.412	1.412	1.412	1.412
**C7-C8**	1.387	1.388	1.388	1.388	1.388	1.388
**C8-C9**	1.402	1.404	1.404	1.404	1.404	1.404
**C9-C10**	1.386	1.387	1.387	1.387	1.387	1.387
**C10-C11**	1.395	1.396	1.396	1.396	1.396	1.396
**C12-C11**	1.448	1.448	1.448	1.448	1.448	1.448
**C12-C17**	1.390	1.390	1.390	1.390	1.390	1.390
**C13-N5**	1.376	1.374	1.373	1.373	1.373	1.373
**C13-C12**	1.415	1.416	1.416	1.417	1.417	1.417
**C14-C13**	1.402	1.403	1.403	1.403	1.403	1.403
**C15-C14**	1.379	1.378	1.378	1.378	1.378	1.378
**C16-C15**	1.411	1.413	1.413	1.413	1.413	1.413
**C16-C17**	1.392	1.393	1.393	1.393	1.393	1.393
**C16-N18**	1.413	1.411	1.411	1.411	1.411	1.411
**N18-N19**	1.242	1.244	1.244	1.244	1.244	1.244
**C20-N19**	1.424	1.424	1.424	1.424	1.424	1.424
**C21-C20**	1.394	1.395	1.395	1.395	1.395	1.395
**C22-C21**	1.387	1.387	1.387	1.387	1.387	1.387
**C23-C22**	1.386	1.387	1.387	1.387	1.387	1.387
**C23-N41**	1.477	1.472	1.471	1.471	1.471	1.471
**C24-C23**	1.390	1.391	1.391	1.391	1.391	1.391
**C25-C20**	1.399	1.400	1.400	1.400	1.400	1.400
**C25-C24**	1.384	1.383	1.383	1.383	1.383	1.383
**N41-O42**	1.212	1.214	1.214	1.214	1.214	1.215
**N41-O43**	1.212	1.214	1.214	1.214	1.214	1.214
**BLA**	0.007	0.008	0.008	0.008	0.008	0.008

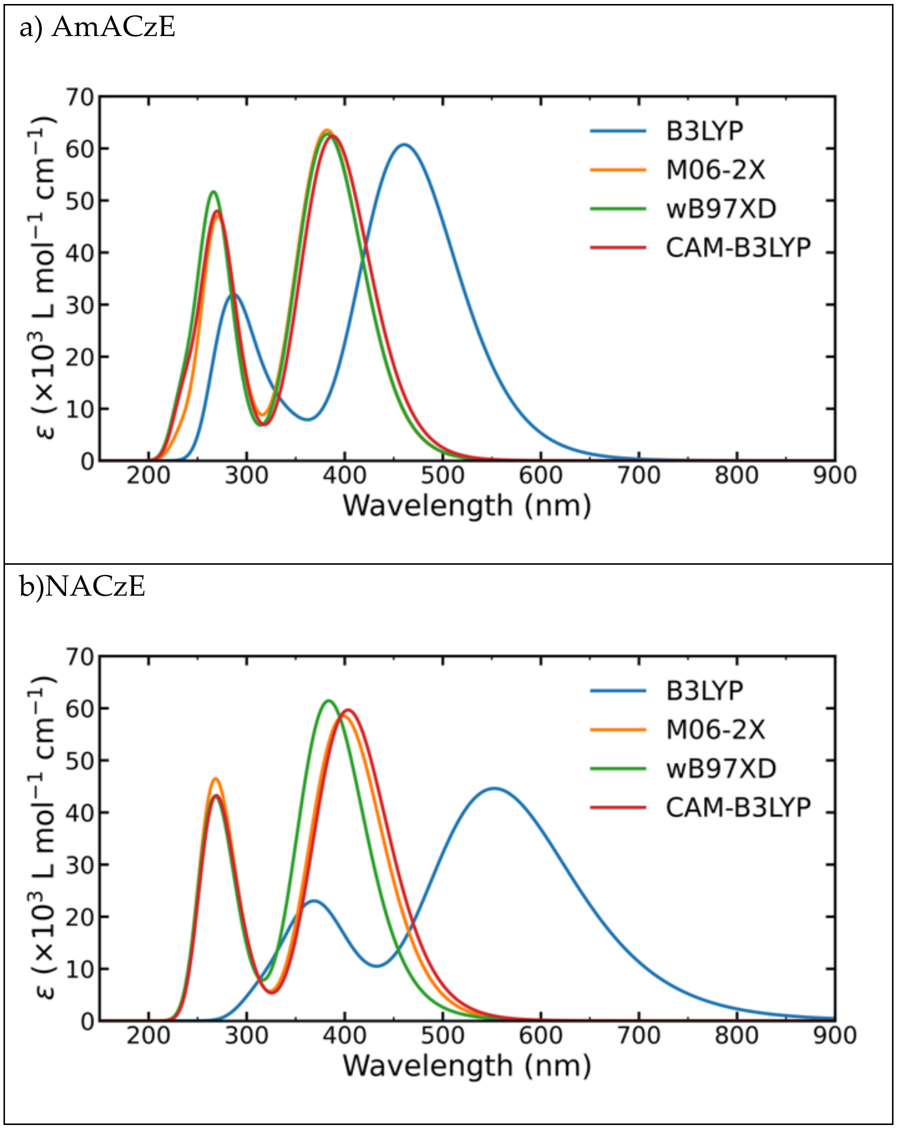

Figure S1. Electronic absorption spectra of AmACzE and NACzE calculated in water (PCM, WAT) using TDDFT with the B3LYP, M06-2X, ωB97X-D, and CAM-B3LYP exchange–correlation functionals in conjunction with the 6-311++G(d,p) basis set. The comparison highlights the systematic underestimation of excitation energies by B3LYP for charge-transfer transitions, while the long-range corrected functional (CAM-B3LYP) provides a more consistent description.

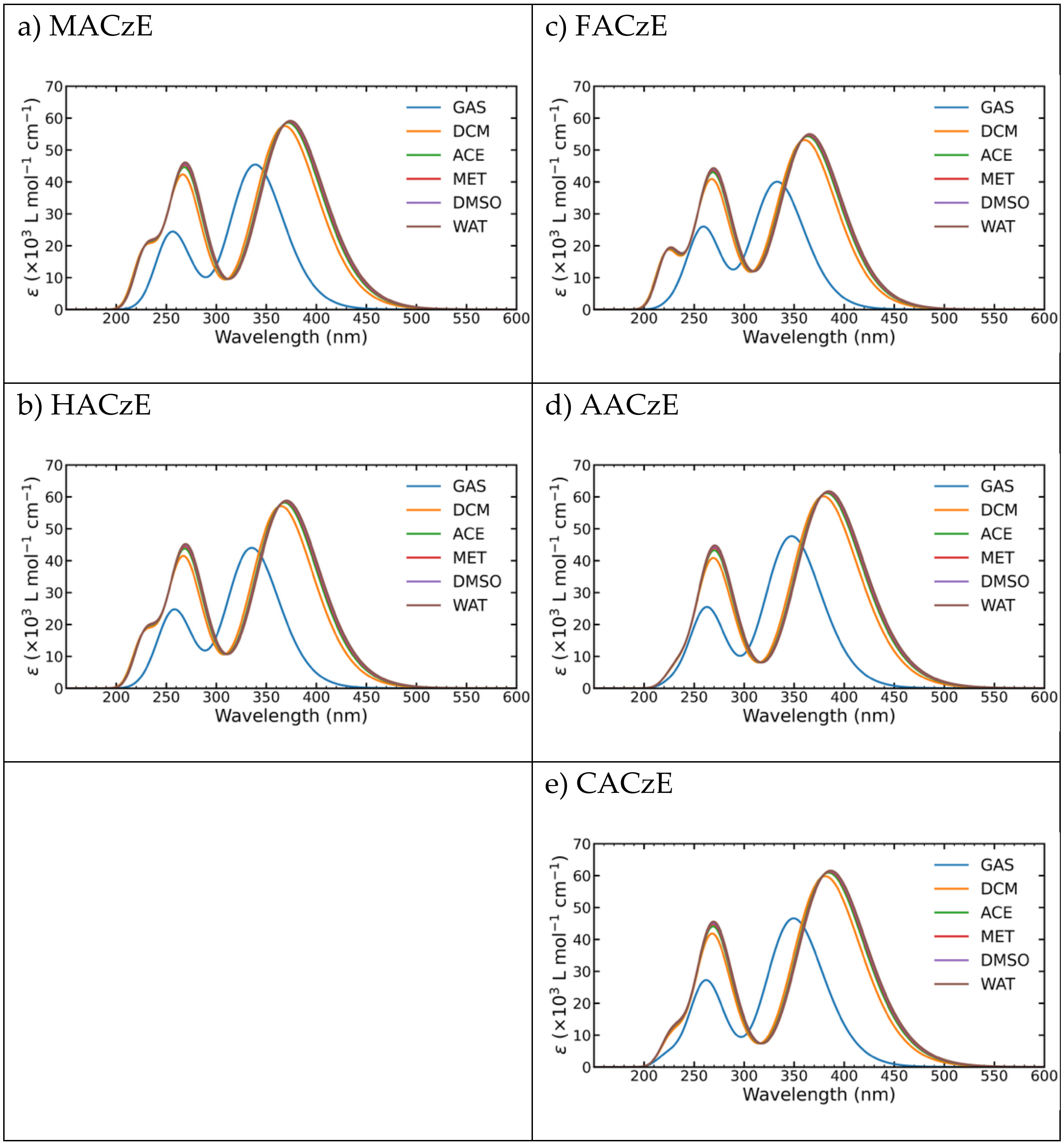

Figure S2. Electronic absorption spectra of (a) MACzE, (b) HACzE, c) FACzE, d) AACzE and e) CACzE calculated at the CAM-B3LYP/6-311++G(d,p) level in the gas phase (GAS) and in different solvent environments: dichloromethane (DCM), acetone (ACE), methanol (MET), dimethyl sulfoxide (DMSO), and water (WAT). These spectra illustrate that the solvent-induced spectral shifts and intensity variations discussed in the main text are systematic across the azo-carbazole series and not limited to the representative examples shown in Figure 2.

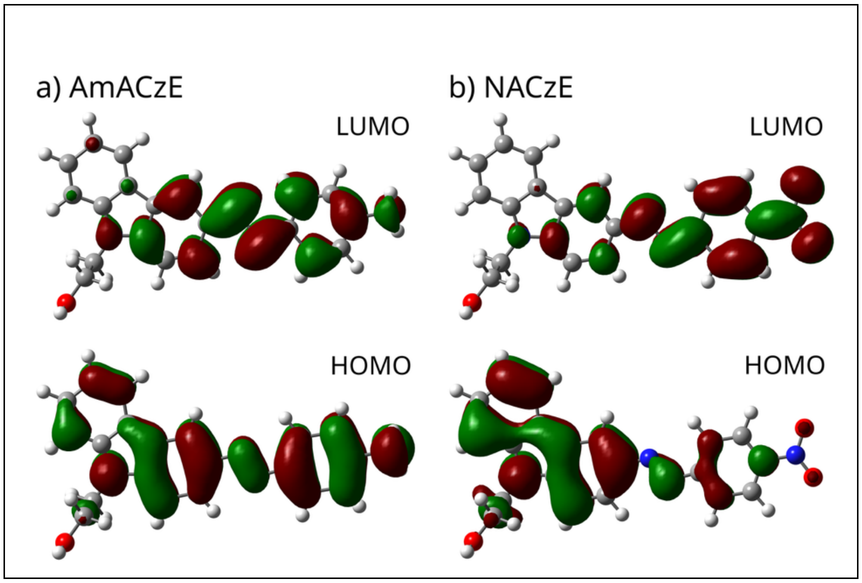

Figure S3. Frontier molecular orbitals (HOMO and LUMO) of AmACzE and NACzE computed at the CAM-B3LYP/6-311++G(d,p) level in water (WAT). The lowest-energy optically allowed transition is dominated by a HOMO→LUMO excitation (hole ≈ HOMO; electron ≈ LUMO), consistent with an intramolecular charge-transfer character driven by the donor–acceptor architecture of the chromophores.

Table S22. DFT Results for Static and Dinamic HRS First Hyperpolarizability ($\beta_{HRS}/10^{-30}$ esu) for AmACzE and NACzE in Water (WAT).

Chromophore	B3LYP	M06-2X	wB97XD	CAM-B3LYP
		Static		
AmACzE	13.03	5.35	5.56	5.53
NACzE	328.54	111.22	90.95	115.16
		λ = 798 nm		
AmACzE	11.58	3.40	3.54	3.46
NACzE	293.45	91.48	73.73	94.48
		λ = 1064 nm		
AmACzE	15.35	3.72	3.87	3.78
NACzE	410.56	106.32	84.40	110.02
		λ = 1313 nm		
AmACzE	22.11	4.17	4.34	4.24
NACzE	683.67	128.75	100.14	133.67
		λ = 1550 nm		
AmACzE	51.74	5.43	5.63	5.52
NACzE	10612.00	196.51	145.38	206.09
